# Non-thermal Plasma Treatment of ESKAPE Pathogens: A Review

**DOI:** 10.3389/fmicb.2021.737635

**Published:** 2021-10-12

**Authors:** Vladimír Scholtz, Eva Vaňková, Petra Kašparová, Ramya Premanath, Iddya Karunasagar, Jaroslav Julák

**Affiliations:** ^1^Department of Physics and Measurements, University of Chemistry and Technology, Prague, Czechia; ^2^Department of Biotechnology, University of Chemistry and Technology, Prague, Czechia; ^3^Nitte University, Nitte University Centre for Science Education and Research, Mangalore, India; ^4^Institute of Immunology and Microbiology, First Faculty of Medicine, Charles University and General University Hospital, Prague, Czechia

**Keywords:** plasma jet, dielectric barrier discharge, corona discharge, biofilm inactivation, bacterial inactivation, antibiotic resistance, antibiofilm activity

## Abstract

The acronym ESKAPE refers to a group of bacteria consisting of *Enterococcus faecium*, *Staphylococcus aureus*, *Klebsiella pneumoniae*, *Acinetobacter baumannii*, *Pseudomonas aeruginosa*, and *Enterobacter* spp. They are important in human medicine as pathogens that show increasing resistance to commonly used antibiotics; thus, the search for new effective bactericidal agents is still topical. One of the possible alternatives is the use of non-thermal plasma (NTP), a partially ionized gas with the energy stored particularly in the free electrons, which has antimicrobial and anti-biofilm effects. Its mechanism of action includes the formation of pores in the bacterial membranes; therefore, resistance toward it is not developed. This paper focuses on the current overview of literature describing the use of NTP as a new promising tool against ESKAPE bacteria, both in planktonic and biofilm forms. Thus, it points to the fact that NTP treatment can be used for the decontamination of different types of liquids, medical materials, and devices or even surfaces used in various industries. In summary, the use of diverse experimental setups leads to very different efficiencies in inactivation. However, Gram-positive bacteria appear less susceptible compared to Gram-negative ones, in general.

## Introduction

The misuse and overuse of antibiotics over the past four decades have not only created exceptional selective pressure toward the emergence of resistant microorganisms but also bestowed the ideal environment for the spread and selection of resistance determinants. The resistance of pathogens to multiple classes of antibiotics is a warning sign and, if not tackled, could bring about the end of the antibiotic era (Boucher et al., 2009). The issue of antimicrobial resistance is one of the greatest challenges faced by humankind, and the Centers for Disease Control and Prevention considers it the biggest threat. The acronym “ESKAPE” (*Enterococcus faecium*, *Staphylococcus aureus*, *Klebsiella pneumoniae*, *Acinetobacter baumannii*, *Pseudomonas aeruginosa*, and *Enterobacter* spp.) was first used in the 2008 to indicate the notorious and life-threatening Gram-positive and Gram-negative bacterial pathogens with the ability to escape or evade killing by antibiotics and to rapidly develop multiple drug resistance (Rice, 2008). ESKAPE pathogens exhibit a wide array of resistance mechanisms, including target site modification, drug inactivation, reduced drug accumulation, and shielding in biofilms (Santajit and Indrawattana, 2016). The infections caused by the ESKAPE pathogens are responsible for high morbidity and mortality, extend hospital stays, and increase treatment costs (Friedman et al., 2016). Globally, it is estimated that antimicrobial resistance would lead to 10 million deaths per year by 2050, which is significantly more than the mortality rates of other diseases such as cancer and diabetes (The PLOS Medicine Editors, 2016).

The impending problem of antimicrobial resistance in ESKAPE pathogens has accelerated the search for new antimicrobials. Antimicrobial resistance and its consequence of a new interest in the development of new antimicrobial therapies were reviewed in a recent paper by De Oliveira et al. (2020). In addition to developing new antimicrobials, novel approaches need to be explored and exploited. These could be found in other scientific fields; for example, physical interference with the viability of microorganisms is a promising procedure with a low threat of development of antimicrobial resistance. Specifically, the use of non-thermal plasma (NTP) may be an efficient tool in the treatment of resistant microbial infection, as has been described many times for microbial inactivation (Moreau et al., 2008; Liao et al., 2017), ready-to-eat food preparation (Scholtz et al., 2015; López et al., 2019), or biofilm degradation (Julák et al., 2018b; Gupta and Ayan, 2019).

This study presents a review of representative publications documenting the impacts of NTP exposure on each member of the high-risk group of ESKAPE pathogens. It is our assumption that this review will be a source of useful information in the research area of nosocomial infections and in the field of plasma medicine.

### Introduction to Non-thermal Plasma

Plasma, the fourth state of matter, consists of partially to completely ionized atoms and the free electrons cleaved from them, exhibiting collective behavior. In general, there are two recognized types of plasma: thermal plasma and NTP, equivalently low temperature and cold plasma. Thermal plasma consists of free electrons and ions that are of the same high temperature of several thousand of kelvins, and it is in a local thermodynamic equilibrium state. Due to this high temperature, it is not applicable in living objects. On the other hand, NTP contains high-energy free electrons and low-energy ions. Because its energy is stored mostly in the light electrons, its overall temperature remains low, mostly approximately 40°C.

**FIGURE 1:**
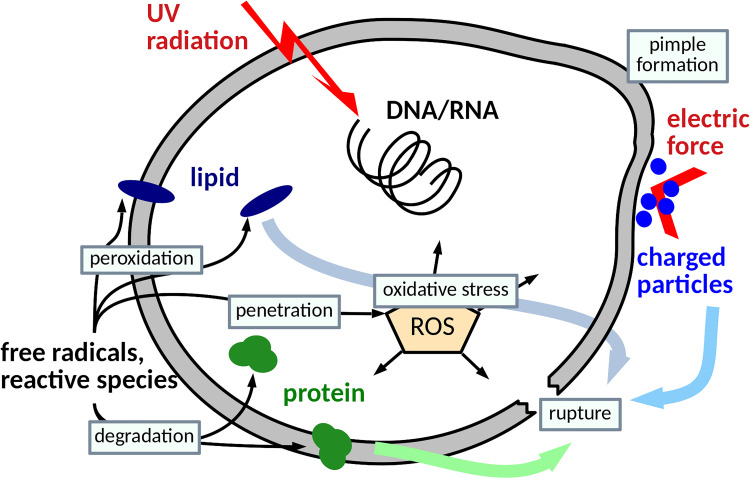
Possible mechanisms of microbial inactivation by non-thermal plasma (NTP).

Both ions and electrons are highly reactive, and their interaction with the surrounding gas or liquids causes chemical reactions and the formation of many highly reactive products (ions, radicals, and particles in unusual electron configurations). In the air, the amounts of reactive oxygen species (ROS) and reactive nitrogen species (RNS) increase (Graves, 2012; Liu et al., 2016). These particles are usually very unstable, with life expectancy in the order of fractions of a second, but also stable particles, especially ozone, hydrogen peroxide, and nitrogen oxides and acids, are formed. The composition and the proportion of these particles are very dependent on the experimental conditions, especially on the parameters of plasma formation and on the composition of the surrounding gas. These particles are responsible for the interaction of plasma with living matter, including the inactivation of microorganisms. Ultraviolet radiation produced to varying degrees by electric discharges is also likely to be involved. The mechanism of the microbicidal action of NTP depends on the actual nature of the plasma-generating discharges, in particular on their geometry, the applied electrical voltage and current, and other experimental parameters. Due to the combination of various mechanisms and chemical reactions, any attempt of the quantification of the NTP dose has not yet been successful and probably is impossible. A detailed description and discussion are beyond the scope of this article. Liao et al. (2017) have described the mechanisms of microorganism inactivation in detail: the basis of these mechanisms is the damage of nucleic acids by UV radiation, lipid peroxidation caused by ROS occurring mainly in fatty acids near the cell surface, and the chemical modification and degradation of proteins caused mainly by hydroxyl radicals. Other studies also reported apoptosis in bacterial cells probably inducted by ROS (Čtvrtečková et al., 2019). Mechanical cell damage, in particular electrostatic disruption caused by the electrostatic forces of charged particles accumulated on the cells, and electroporation by the direct bombardment of charged particles also applied. For illustrative purposes, simplified inactivation mechanisms are shown in Figure 1. In the majority of reports, Gram-negative bacteria showed higher sensitivity to NTP than did Gram-positive ones. This is probably caused by the stable and thick peptidoglycan layers of Gram-positive bacteria as opposed to those of Gram-negative bacteria (Liao et al., 2017). However, this cannot be considered as a general fact, and the actual sensitivity may depend on the particular bacterial strain, as shown, e.g., in Paldrychová et al. (2019). This study reported that the biofilms of *P. aeruginosa* clinical isolates were not eradicated, while the collection strains were eliminated almost completely.

Non-thermal plasma with convenient microbicidal effects has potential for practical application due also to its several interesting features, such as on-demand operation (easy on/off), low cost and simple handling, sporadic maintenance, performance at ambient temperature and atmospheric pressure, and ability to act in dry conditions. The gaseous nature of NTP and the produced neutral particles facilitates access to highly confined spaces and penetration into micro- or nanoporous materials. The combination of several described physical and chemical mechanisms also reduces the possible evolution of bacterial resistance to a minimum. Regarding the disadvantages or limitations of NTP application, the following should be mentioned: it affects the properties of the exposed surfaces of solid objects or on the upper layer of liquids, it has a non-remote action (unlike UV radiation), and it is impossible to store.

An interesting effect is that water and aqueous solutions exposed to plasma retain their microbicidal activity for a long time, up to several months. The activity of plasma-activated water (PAW) is caused by the persistence of stable microbicidal compounds, especially hydrogen peroxide, nitrogen acids, and, probably, also ozone (Laurita et al., 2015; Hozák et al., 2018; Julák et al., 2018a; Thirumdas et al., 2018).

### Generation of Non-thermal Plasma

There are several possible ways of generating NTP, although, for its microbicidal effects, this is generally achieved by means of electrical discharges in various configurations. Their most common arrangements are briefly described below and in more detail in Ehlbeck et al. (2011) and Šimončicová et al. (2019).

Corona discharges are generated by applying a DC voltage of several kilovolts to a sharp tip, blade, or thin wire in positive or a negative configuration of electrodes. For positive corona in the needle–plate electrode configuration, discharges start with burst pulse corona and proceed to the continuous (Bastien and Marode, 1979) or streamer corona, glow corona, and spark discharge as the applied voltage increases (Chang et al., 1991). For negative corona, the initial form is the Trichel pulse corona, followed by continuous corona and spark discharge as the applied voltage increases. Machala et al. (2005) described the stabilization of the transition of continuous discharge to the spark or arc using an appropriate ballast resistor. Khun et al. (2018) described the discharges called curved transition spark, interrupted channel, “baby doll,” and branched transition spark. The special arrangement of the spike electrodes creates a flame-like so-called cometary discharge, resembling a plasma jet (Scholtz and Julák, 2010; Scholtz et al., 2013).

Plasma jet (also plasma needle, plasma torch, and plasma pen) is probably the most commonly used type of source at present. Plasma arises here in the active region of the used discharge and is blown by flowing auxiliary gas (Ar, He, air, and others), usually at atmospheric pressure. The gas pulls the particles outside the electrode area in propagating ionization waves and forms a stream of active particles burning as a small jet. The advantages of jets are their uncomplicated design and easy maintenance; like corona discharges, however, they only reach a relatively small target area (Laroussi and Akan, 2007; Nishime et al., 2017). The Leibniz Institute for Plasma Science and Technology (INP Greifswald, Germany) has developed a range of plasma jet devices. Under the designation kINPen (kINPen^®^ MED, kINPen 11, kINPen09^®^, etc.), these are supplied by Neoplas tools GmbH (Greifswald, Germany).

Dielectric barrier discharge (DBD) arises when the AC voltage is applied to two flat metal electrodes separated by a non-conductive dielectric (glass, plastics, etc.). The dielectric avoids the charge transport, and the discharge burns due to the electric induction and the polarization of dielectric only. The advantage of this arrangement is the larger exposed area; the disadvantage is the small distance of several millimeters between the electrodes. Detailed description and a comparison of DBD with corona discharges have been published (Yehia, 2019). The start-up company Plasmapp (Daejeon, South Korea) launched STERPACK, which is based on DBD and declared as an effective method of biofilm removal from plastics and foods (Lee, 2018).

Microwave discharges are usually generated in a box by the resonance of high-frequency (hundreds of megahertz) electromagnetic waves. This arrangement was often used in basic research on plasma–biomaterial interactions and was already certified for medical use (Isbary et al., 2013). Several manufacturers supply microwave sterilizers working at reduced pressure and/or in the atmosphere of hydrogen peroxide as additional disinfecting agents. Among them, the Sterrad^®^ series (Johnson & Johnson, New Brunswick, NJ, United States) and PE-200 (Plasma Etch, Inc., Carson City, NE, United States) should be mentioned. The Plazlyte System (AbTox Inc., Mundelein, IL, United States), using peroxyacetic acid as an additive, was promising, but the Food and Drug Administration did not recommend its routine use. These pieces of equipment work at ambient temperature and are therefore suitable for the sterilization of temperature- or corrosion-susceptible instruments, such as endoscopes. They can thus replace sterilization in autoclaves or with toxic ethylene oxide. Bryce et al. (1997) and Adler et al. (1998) presented evaluations of these instruments.

## Non-Thermal Plasma Treatment of Eskape Pathogens

The literature has already acknowledged the great potential of the antibacterial effect of NTP on the ESKAPE pathogens, with many publications dealing with this topic. Most such publications are shown in Supplementary Tables 1–6, which detail the bacterial strains used and the properties of NTP exposure (discharge, output voltage, frequency, and gas used) together with a brief summary of their antimicrobial efficacy [given as log reductions in colony forming units (CFU) per milliliter], inhibition zone, and inhibition in relative percentages. Concerning the antimicrobial efficacy of NTP exposure, the results are vast and are difficult to shorten into concise text. The following review is divided into subsections focused on particular bacterial species and subdivided by the forms of exposed bacteria: planktonic or surface growth, biofilm formation, and other special applications.

In general, the total inhibition of planktonic ESKAPE growth is achieved by the exposure of cells to NTP from 1 min to 3 h, depending on the device, type of discharge, output voltage, etc. The DBD sources are, on average, efficient after tens of minutes of exposure, but significant inhibition can be achieved even after a shorter time. Plasma jets usually achieve complete inhibition after several minutes, but have to be focused on small areas only. Corona discharge devices operate at similar times to DBD. However, the results differ with every study published due to many variables, which affect the efficacy of NTP treatment.

Despite NTP treatment being well documented for planktonic cells, it must be noted that most microorganisms exist in the environment in a form of biofilm. The penetration of antibiotic drugs into biofilms is retarded by the biofilm matrix, and the cells in biofilms often adapt to very slow metabolisms, which makes them less sensitive to antibiotic treatment. Taken together, biofilms are very difficult to be eradicated using common antimicrobial approaches, which, again, makes NTP a promising anti-biofilm agent.

A completely different application of NTP is based on its indirect effect *via* PAW or other plasma-activated solutions (e.g., buffers and gels). In the stated case, NTP is used strictly for the treatment of liquid without the presence of microbial cells. The exposure creates reactive species in the treated liquid, which is, thanks to them, responsible for the antimicrobial effect when mixed with the cells of pathogens. The efficiency of PAW is dependent on the time of NTP exposure, the storage temperature, time of storage before treatment, and the actual treatment time of cells.

Before we begin to review the particular bacterial species, thorough studies (Flynn et al., 2015; Modic et al., 2017) devoted to the whole group of ESKAPE pathogens should be mentioned. In Flynn et al. (2015), helium (99.5%) and oxygen (0.5%) atmospheres were used. Standard plate counts and the metabolic assay were used to evaluate the antibacterial effects, which showed comparable eradication times. Rapid antimicrobial effects against all the ESKAPE pathogens in the planktonic form of growth were found. *Enterobacter cloacae* was the most susceptible to NTP exposure, with complete eradication achieved within 45 s. *P. aeruginosa* was completely eradicated within 60 s, *E. faecium*, *K. pneumoniae*, and *A. baumannii* were completely inactivated within 120 s of NTP exposure, and *S. aureus* was the most resistant, requiring 240 s of NTP exposure. The complete eradication of the ESKAPE pathogens in biofilm form was achieved within 360 s, except for the resistant *A. baumannii*. On the other hand, *K. pneumoniae* appeared as the most sensitive, being inactivated within 120 s. The other study of Modic et al. (2017) has shown the possibility of inactivating *K. pneumoniae* and other ESKAPE pathogens in mixed biofilms. Two models of discharge were compared: a low-power discharge, giving plasma dominated by ROS, and a high-power discharge, giving plasma dominated by RNS. Using RNS, the bacteria in individual biofilms were killed rapidly, with no survivors after 15 s of NTP exposure. *S. aureus* survived longer under these conditions, with no detectable growth after 60 s of exposure. Using RNS in mixed-species biofilms, *P. aeruginosa* survived longer, but all species were killed with no detectable growth at 60 s. On the other hand, using ROS, all pathogens including *K. pneumoniae* showed viable cells after 240 s of exposure, with *P. aeruginosa* showing significant survival.

### *Enterococcus faecium* and *Enterococcus faecalis*

*Enterococcus* spp. are Gram-positive facultative anaerobic cocci belonging to the regular and beneficial component of the intestinal microbiota. They also occur in the vagina, the oral cavity and teeth, and, rarely, in the upper airways. The species *E. faecium* and *Enterococcus faecalis* predominate in the human intestine and thus are frequent ingredients of probiotics. *E. faecium* has been previously described as a commensal of the gut with little medical importance. However, it has afterward emerged as one of the important nosocomial pathogens causing urinary tract infections, especially in patients with long-term urinary catheters, anatomical abnormalities, or as complications of surgical procedures. Endocarditis in patients with heart disease and cardiac valve replacement, abdominal and pelvic infections with abscess formation in patients after peritoneal dialysis, and bloodstream and wound infection, among others, also occur (Simmons and Larson, 2015). Resistance of *Enterococcus* spp. to antibiotics is very important as they have great ability to exchange genetic material and quickly acquire new types of resistance that they can pass on to other bacteria. *E. faecium* elaborates several virulence factors, including hyaluronidase, gelatinase, aggregation substance, cytolysin, and enterococcal surface protein, as well as biofilm formation (Upadhyaya et al., 2009). In a review of biofilm-related organisms that infect medical devices implanted in a human body, Donlan (2001) listed those usually colonized by *Enterococcus* spp., as follows: central venous catheters (*E. faecalis*), prosthetic heart valve (*Enterococcus* spp.), urinary catheters (*E. faecalis*), artificial hip prosthesis (*Enterococcus* spp.), and intrauterine devices (*Enterococcus* spp.). *E. faecium* is intrinsically resistant to penicillins, cephalosporins, aminoglycosides, and sulfonamides (Crank and O’Driscoll, 2015). It has acquired resistance to various other antimicrobial agents, including macrolides, glycopeptides, and oxazolidinones, through horizontal gene transfer (Arias and Murray, 2012). The World Health Organization (WHO) has designated the vancomycin-resistant *Enterococcus* spp. (VRE) as high-priority pathogens due to their limited therapeutic options (Tacconelli et al., 2018). Infection with VRE resulted in over three times hospital and antibiotic costs to the patients (Zhen et al., 2019). Six types of VRE have been reported (VAN-A to VAN-E and VAN-G), with VAN-A being mostly associated with hospitalization and showing resistance to glycopeptide antibiotics (Santajit and Indrawattana, 2016).

Due to their pathogenicity, *Enterococcus* spp. are a frequent subject of a lot of research and modeling and clinical studies of their interaction with NTP. The two most important pathogens, *E. faecium* and *E. faecalis*, are closely related and have comparable properties. Although they can be distinguished by a simple mannitol fermentation test (Quiloan et al., 2012), they are often referred to without distinction as *Enterococcus* spp. For this reason, we described the effects of NTP on both species in this section and present a summary in Supplementary Table 1.

#### Planktonic Form

According to the available literature, the planktonic form of *Enterococcus* spp. was applied on diverse types of solid surfaces or carriers and exposed to NTP in a variety of experimental setups. Daeschlein et al. (2010) tested the action of argon NTP toward suspension of typical wound infection-associated pathogens, including *E. faecium*, inoculated on agar surface imitating wound colonization. The monitored microorganisms on agar media were exposed to NTP three times for up to 2 min (with this experimental setup, a contaminated area of 55 cm^2^ was treated completely within 6 min). The NTP efficacy was generally almost as strong as the power of antiseptics; however, the lowest reduction factor (RF = 1.9) was obtained only for *E. faecium* (initial concentrations of 10^2^–10^3^ log CFU/plate). In a subsequent study, Daeschlein et al. (2014) compared 194 tested clinical wound pathogens from 13 varied species including *E. faecium* and *E. faecalis*. After 3 s treatment, they observed the inhibition zones, which differed from species to species and showed the higher susceptibility of Gram-positive bacteria. The largest zones were observed for enterococci, with inhibition areas of 480 and 20 mm^2^ for DBD and plasma jet, respectively. Ermolaeva et al. (2011) inactivated the clinical isolates of seven pathogenic bacterial species, including *E. faecium*, inoculated on agar plates in a concentration range of 10^3^–10^5^ CFU/plate. Overall, the Gram-negative bacteria appeared to be more susceptible to argon NTP treatment than did the Gram-positive bacteria. *E. faecium* displayed medium resistance. A 10-min treatment completely suppressed bacterial survival on agar plates. Also, Scholtz et al. (2010) compared the effects of NTP on various microorganisms grown on agar plates. In accordance with the previously mentioned publication, *E. faecium* proved to be moderately sensitive: inoculated on agar in the 10^6^-CFU/cm^2^ concentration and exposed to NTP, it provided a 38-mm^2^ inhibition zone of a circular shape free of any surviving bacteria. Nishime et al. (2017) also observed an inhibition zone of 100 mm^2^ after several minutes of exposure. Lis et al. (2018) treated *E. faecium*, *K. pneumoniae*, *A. baumannii*, and other bacterial suspensions at an initial concentration of 10^6^ CFU/cm^2^ dried on stainless steel surfaces (3.14 cm^2^) with NTP for 5, 10, and 20 min. A 3.66 log reduction was achieved for *E. faecium* after 20 min of exposure. Comparable results were shown for the other ESKAPE pathogens tested without any significant differences between the representatives of Gram-positive and Gram-negative bacteria. In general, bacterial cells covered with bovine serum albumin (BSA) for the simulation of natural organic material were significantly less inactivated in comparison with the cells in phosphate-buffered saline (PBS) suspension. In the study of Klämpfl et al. (2014), an even higher sensitivity (a 3 log reduction) of bacterial endospores (*Clostridium difficile*) on a stainless steel carrier exposed to NTP for 10 min was observed in comparison with vegetative bacteria including *E. faecium* (only a 2 log reduction). Finally, Chen et al. (2012) designed an NTP source for large area sterilization. Helium and oxygen were used as the working gas. The helium/oxygen NTP killed *E. faecalis* more effectively than did the pure helium NTP. As determined by optical emission spectroscopy, reactive species such as O and OH radicals were probably responsible for the sterilization effect.

#### Biofilm

Jiang et al. (2012) verified the hypothesis that *E. faecalis* biofilm may be inactivated by NTP *in vitro*. An *E. faecalis* monoculture biofilm was cultivated on the surface of hydroxyapatite disks for 6 days and exposed to NTP and/or 5.25% sodium hypochlorite (NaOCl) solution. The CFU counts and scanning electron microscopy (SEM) showed that treatment of the *E. faecalis* biofilm with NTP and NaOCl for 5 min resulted in 93 and 90% reductions, respectively. Nearly no intact bacteria were discernible after NTP exposure. Similarly, Theinkom et al. (2019) studied the effect of NTP on *E. faecalis* inoculated on agar plates or on its biofilm form. The 5- and 10-min exposures of 24-h-old biofilms resulted in more than 3 and more than 5 log reductions in CFU/plate, respectively. In biofilm experiments, chlorhexidine (CHX) and UV-C radiation were only slightly more effective than NTP. Moreover, there was no damage of the cytoplasmic membranes upon NTP treatment; thus, in this case, it seems that membrane damage is not the primary mechanism of NTP action. In another study, Cao et al. (2011) evaluated the efficacy of NTP against *E. faecalis* and *S. aureus* suspensions on agar plates and *E. faecalis* biofilm on nitrate membrane filters. In agar cultures, inhibition zones were observed after NTP treatment; the diameter of the zones for both bacteria increased with prolongation of the treatment duration. However, ultrastructural changes in the *E. faecalis* biofilm were observed by electron microscopy after 2 min treatment. It was concluded that NTP could serve as an effective supplement to standard endodontic microbial treatments.

#### Biofilm in Root Canals

*Enterococcus faecalis* is one of the most important bacteria causing failure of root canal treatments; thus, many studies have been devoted to this problem as well. As a fundamental review in this field, the one by Hoffmann et al. (2013) should be mentioned. The important methods of NTP production are listed and described therein, namely, DBD, atmospheric pressure plasma jets, radio frequency plasma jets, pulsed direct current-driven plasma jets, plasma needle, and plasma pencil. Among the working gases, the use of helium, argon, nitrogen, a helium and oxygen mixture, and air is recommended. State-of-the-art lab-scale research and clinical trials of NTP in stomatology were reviewed by Li et al. (2017).

Du et al. (2012) tested the efficiency of NTP and antiseptic CHX against *E. faecalis* biofilm formed on coverslips and in root canals simulating the conditions during endodontic therapy. NTP treatment was comparably effective to CHX for bacteria inactivation in infected root canals after 5 and 15 min exposures (both agents with 6 log decreases in CFU/ml). The results were confirmed using confocal laser scanning microscopy (CLSM) and micro-computed tomography. In their follow-up study, Du et al. (2013) focused on the investigation of the actions of NTP and CHX on *E. faecalis* and multispecies (mixture of bacteria from human dental root canal infections) biofilms grown on bovine dentin disks. Biofilms (1 and 3 weeks old) were exposed to NTP, modified non-equilibrium NTP with CHX, and CHX for 2 and 5 min. The exposed samples were examined with CLSM and 3D reconstruction analysis. No significant difference was detected between the actions of NTP and CHX. Modified NTP with CHX was the most effective in killing bacteria; significantly more cells were killed in 1-week-old biofilms than in 3-week-old biofilms. Simoncelli et al. (2015) described the dental applications of NTP against *E. faecalis* on Tryptone Soya Agar plates, quantitatively evaluated on bacterial suspensions and tested in root canals of extracted teeth. The direct treatment of root canals in a dry environment was shown to cause the highest level of bacterial inactivation, but a relevant bacterial load reduction was also obtained when the root canal system was irrigated with PAW. Hüfner et al. (2017) aimed to compare the effect of NTP with NaOCl and to use their combination on *E. faecalis* biofilm formed in root canals of extracted human teeth. It was shown that the mutual treatment with NaOCl and NTP has an additive effect (but not statistically significant) in the log CFU reduction of *E. faecalis* biofilm as compared to NaOCl monotherapy. The results were also confirmed using SEM. Zhou et al. (2016) used NTP with helium working gas bubbled through 3% hydrogen peroxide to treat the root canal infected for 1 week with *E. faecalis*. This experimental setup was also compared with ones without hydrogen peroxide. The greatest reductions in CFU/ml (a 7 log decrease) were obtained after 4 min exposure of NTP with helium bubbled through hydrogen peroxide. Regarding the disinfection mechanism, atomic oxygen and hydroxyl radicals are considered responsible for the improved antibacterial effects. Li et al. (2015) treated endodontic *E. faecalis* biofilm (formed for 3 weeks in root canals) with argon/oxygen NTP (12-min exposure) and compared it with those treated with calcium hydroxide, 2% CHX gel, and calcium hydroxide/CHX for 1 week. The most effective treatment was the NTP exposure; there were no detectable bacteria alive after 12 min of NTP exposure, as confirmed by CFU counting, SEM, and CLSM. The microhardness and roughness of root canal dentin showed no significant difference after NTP treatment. Pan et al. (2013) treated a set of single-root teeth infected with *E. faecalis* biofilm. NTP treatment for 8 and 10 min had a significant anti-biofilm efficacy with a total reduction in CFU/sample. SEM and CLSM showed that the bacterial membrane was ruptured and the structure of the biofilm was fully destroyed. Finally, the study by Üreyen Kaya et al. (2014) compared the antimicrobial efficacy of NTP with a gaseous ozone delivery system and NaOCl solution rinsing on *E. faecalis*-contaminated root canal walls and dentine tubules performed on extracted human mandibular premolars with straight root canals. A superior efficacy of NTP compared with NaOCl was demonstrated. Both NaOCl and NTP were better than ozone at the coronal and middle parts of the root canals.

#### Plasma-Activated Water

The idea of PAW treatment was improved by Ercan et al. (2013), who tested the antibacterial effect of 100 mM PAW solution of *N*-acetylcysteine (NAC) against planktonic cells and biofilm of various bacteria. The total inactivation of 10^7^ CFU/ml of *E. faecalis*, *S. aureus*, *A. baumannii*, and *P. aeruginosa* in both planktonic and biofilm forms was observed after 15 min of application. In a follow-up work, Ercan et al. (2014) demonstrated a low but significant inhibitory activity of plasma-activated solutions of methionine, threonine, glucose, cysteine, proline, glycine, glutamine, heparin, and arginine on *E. faecalis*, *A. baumannii*, *S. aureus*, and *K. pneumoniae*.

### Staphylococcus aureus

*Staphylococcus aureus*, a Gram-positive coccus, is commonly found in moist areas such as the nasal cavity and the armpits of healthy individuals. Approximately 20% of individuals who always carry a single strain of *S. aureus* are known as persistent carriers; 60% of individuals are called intermittent carriers, as they harbor *S. aureus* occasionally. *S. aureus* belongs to so far the most severe nosocomial pathogens of today’s medicine, causing a wide spectrum of diseases and pathological states either by direct infection of the host organism or indirectly *via* the production of various virulence factors (Tong et al., 2015). The very first organism shown to produce biofilms was *S. aureus*. Since then, biofilms produced by *S. aureus* are recognized as the leading cause of chronic infection in patients with implants such as heart valves, orthopedic devices, and shunts (Khatoon et al., 2018). The virulence of *S. aureus* is mediated by many extracellular proteins. Nearly all strains of *S. aureus* elaborate collagenase, hyaluronidase, hemolysins, and proteases, thus helping in the primary establishment of infection by breaking down the tissue (Boubaker et al., 2004). Nearly 25% of *S. aureus* strains produce toxic shock syndrome toxin 1 (Parsonnet et al., 2005). Leucocidin, a pore-forming exotoxin emanated by less than 5% of *S. aureus* strains, is involved in causing severe necrotic hemorrhagic pneumonia (Vandenesch et al., 2003). Staphyloxanthin of *S. aureus* acts as an antioxidant and sequesters ROS produced by neutrophils, thus helping the bacterium survive (Clauditz et al., 2006). Staphylococcal infections are comprehensively described in many publications and thus are not going to be described in depth in the present review. Briefly, *S. aureus* can directly cause sepsis, endocarditis, chronic infections from colonized medical devices, bloodstream infections, and respiratory diseases, which, in immunocompromised patients, can easily lead to death. Indirectly, *S. aureus* is responsible for food poisoning, toxic shock syndrome, and scalded skin syndrome, which is considered to be life-threatening, or other various dermatitis (Stevens et al., 2002; Monecke et al., 2011). Methicillin-resistant *S. aureus* (MRSA), initially described in the 1960s, is any strain of *S. aureus* that is resistant to all penicillins, cephalosporins, and carbapenems (Smith, 2004). At present, infections with MRSA are a major health concern throughout the world. Although glycopeptides such as vancomycin have been used against MRSA, resistance has been emerging, and *S. aureus* resistant to other non-β-lactams including vancomycin has compounded the health issues (Cong et al., 2020). Vancomycin resistance in *S. aureus* is encoded by the *van-A* gene also found in VRE, and many MRSA strains carry both *van-A* and *mec-A*, indicating genetic exchange with *E. faecium* (Appelbaum, 2007). The reported incidence of MRSA-related infections is very high (7–60% in the United States) (Siddiqui and Koirala, 2018). With a large number of people infected yearly with this bacterium, the need for efficient treatment is relevant. Because the mechanism of NTP action is strikingly different from those of common antibiotics, resistance of *S. aureus* cells toward NTP is very unlikely to develop, which makes treatment very promising in today’s research and medical practice.

This bacterial genus is probably the most studied in the field of NTP antimicrobial and anti-biofilm activity. Most of the studies are clearly summarized in Supplementary Table 2. In the following section, selected interesting publications are described in more detail.

#### Planktonic Form

In planktonic form, Burts et al. (2009) observed a 4 log reduction in CFU/ml after exposure of *S. aureus* inoculated on agar plate to NTP for 10 min. On the other hand, Yong et al. (2019) tried to disinfect *S. aureus* inoculated on pork jerky pieces exposed to NTP for 30 min, but achieved only a 1 log reduction in the CFU counts. Usta et al. (2019) exposed a cell suspension of *S. aureus* for 15 min, which resulted in only a 0.25 log reduction in CFU/ml. The increment of the treatment time to 30 min resulted in total inhibition, indicating a frequent leap effect, where NTP acts very mildly for long time and then suddenly kills all cells with the increment of exposure time. Liao et al. (2018b) experimented with the adaptation of cells to various physical stresses such as increased acidity, osmotic pressure, and temperature. In this case, non-adapted cells of *S. aureus* were reduced by 7.1 log CFU/ml after 30 s exposure to NTP. The acidity-adapted cells were even more susceptible toward NTP treatment, with the same exposure time resulting in a 7.5 log reduction in survival counts. On the other hand, when the cells were adapted to osmotic pressure, heat or cold, their susceptibility dropped significantly, and the same NTP treatment resulted in a lower reduction of only 3 log CFU/ml. Such study indicates how environmental properties affect the cell persistence of *S. aureus*, which is relevant especially in the food industry for the proper packaging and storing of food. Its importance also lies in humidity of the surrounding atmosphere, as described by Ki et al. (2019). They examined the effects of 20–50% and 70–90% humid air on the efficacy of NTP exposure in *S. aureus* cell suspension inhibition. The low humidity of air resulted in only a 1.5 log reduction in CFU/ml after 60 min of NTP exposure, while the high humidity of air completely inhibited cell growth. The increased efficiency of such NTP exposure is obviously caused by the different proportions of reactive species formed, with water-originated radicals as the most prominent. Such finding would benefit the NTP decontamination of wet food samples, although its effect on the sensory properties of treated food must be thoroughly studied. Food contamination by *S. aureus* was also studied by Choi et al. (2018), who focused on paprika powder loaded with drops of *S. aureus* suspension. The authors used multiple-cycle treatments for only 2 min with various power settings of NTP. After a five-cycle treatment with 1.5 kW power, there was only a 2 log reduction in CFU/ml. They then combined NTP treatment (2 min, 1 kW) with radio frequency heating (2 min, 1.5 kW) for two cycles, which resulted in the complete inhibition of *S. aureus* on paprika powder. The combination of NTP exposure with other physical treatments was also proven to be effective by Liao et al. (2018a), who treated the *S. aureus* cell suspension with NTP for 5 min and subsequently with ultrasound for 10 and 20 min. The subsequent ultrasound treatment for 10 min resulted in a 3.8 log reduction in CFU/ml, which was greater than that with NTP acting alone (2.5 log reduction). The combination of 5 min NTP exposure with 20 min of ultrasound treatment resulted in complete inhibition.

Zhang et al. (2017) exposed a cell suspension of *S. aureus* to 7 min of NTP and achieved complete inhibition of growth. In this study, they also tried to decrease the exposure times; the 5-min treatment resulted in only a 3.4 log reduction. Comparable results were also achieved by other researchers, with treatment times ranging from 2 to 4 min needed for prevalent and complete inhibition (Guimin et al., 2009; Alkawareek et al., 2014; Flynn et al., 2015; Parkey et al., 2015; Yoo et al., 2015; Lunov et al., 2016a).

An interesting study by Vaze et al. (2017) evaluated the effect of NTP on bioaerosol of cell suspension created by nebulizer and achieved total suppression in the CFU/ml counts, indicating that treatment of very few nebulized drops of liquid might be a very effective form of therapy.

#### Biofilm

The pathogenicity of *S. aureus* is mostly facilitated by its ability to form compact biofilms on both biotic and abiotic surfaces. The occurrence of biofilm-related *S. aureus* infection is very common, so it is not surprising that NTP is primarily used to eradicate such contamination or to prevent surface biofilm formation. Miletić et al. (2014) focused on the treatment of cell suspension before the biofilm formation in polystyrene microtiter plates using NTP discharge working at two power settings. Total inhibition of biofilm formation (a 5 log decrease in CFU/ml) was achieved either by 2-min treatment with NTP produced at 0.9 W or only a 1-min exposure to NTP formed at 1.6 W. Different power regimes for NTP formation were also used in Modic et al. (2017). The NTP used in this study achieved total inhibition (6.5 log decrease) of biofilm formation on coupons after 2 min at both working powers (8 and 34.5 W), similarly to Miletić et al. (2014). Cotter et al. (2011) studied the effect of NTP on *S. aureus* biofilms formed on coverslips. NTP exposure for 1.5 h resulted in 90% inhibition in biofilm cell viability as quantified by the MTT (3-[4,5-dimethylthiazol-2-yl]-2,5 diphenyl tetrazolium bromide) viability assay. A similar result was also achieved in the study of Usta et al. (2019). NTP exposure caused an 86% inhibition of the viability of *S. aureus* biofilm cells after 2 h of treatment. Interestingly, treatment of only 30 min resulted in the total suppression of cell survival as determined by CFU/ml counting (5 log decrease), which only highlighted the problematic evaluation of biofilms and the effects of various antimicrobial agents on them. Alkawareek et al. (2014), also mentioned for *P. aeruginosa*, eradicated the *S. aureus* biofilm pre-formed on the peg lid of the Calgary Biofilm Device after several minutes.

Since biofilms are complex structures consisting of both cells and the extracellular matrix in between them, Xu et al. (2015) studied the efficacy of NTP exposure in killing *S. aureus* biofilm with LIVE/DEAD staining on CLSM. They found that the first to the fourth layer (0–5 μm) of biofilm cells were indeed killed, but the deeper layers (6.2–10 μm) showed only partial cell killing. The layers close to base (11–18.5 μm) of the biofilm were completely unharmed. Ferrell et al. (2013) also studied the effect of NTP on the properties of *S. aureus* biofilm. Using COMSAT analysis (a routinely used image analysis of CLSM z-stack pictures), they evaluated the biofilm porosity, thickness, biovolume, and roughness, among other properties. After all exposure times used, the biofilm volume decreased and the porosity and roughness increased, indicating damage of the upper biofilm layers, which correlates with the previously mentioned study. Czapka et al. (2018) also studied the effect of NTP on biofilm morphology and found that NTP exposure resulted in the increased porosity and roughness of the formed biofilm. Interestingly, they also found that, in comparison to *S. aureus*, *Escherichia coli* was less susceptible toward NTP, and its biofilm formation was not inhibited or even slightly affected.

#### Plasma-Activated Water

Xiang et al. (2019) prepared PAW for only 1 min and mixed it with the cell suspension of *S. aureus* for 6 min. The treatment resulted in a 2.3 log reduction in CFU/ml. Laurita et al. (2015) prepared PAW for longer times (5 and 10 min). PAW exposed to NTP for 5 min resulted, after 25 min treatment of *S. aureus* cells, in a 6.5 log reduction in CFU/ml. When the prolonged time of PAW preparation (10 min) was used, the same reduction was already achieved at 15 min treatment of suspension cells. Ma et al. (2015) used PAW exposed for 10 and 20 min for the decontamination of *S. aureus*-loaded strawberries. PAW prepared for 10 min resulted, after 15 min treatment, in a 1.6 log reduction of CFU/ml, while PAW prepared with 20 min NTP exposure caused a 2.3 log reduction after the same amount of time. Schmidt et al. (2019) used an even longer NTP exposure time (30 min) in the preparation of PAW, which, after a 30-min treatment of *S. aureus* cells, completely suppressed cell survival. Chen et al. (2018) used the same exposure time for the preparation of PAW; after 3 h of cell treatment, this resulted in only a 2.3 log reduction in CFU/ml. Interestingly, such findings were also complemented with information on the metabolic activity of the treated cells reflecting the actual viability. Although the CFU/ml reduction was not complete, most cells were metabolically inactive (99% in comparison with the control samples) according to the MTT viability assay. Such lack of correlation between the cell viability and culturability was also found in other studies, which, however, used the direct NTP effect (Cotter et al., 2011; Flynn et al., 2015; Lee et al., 2015; Seo et al., 2017; Czapka et al., 2018). This reflects that, although most cells were metabolically inactivated after relatively short NTP exposure times, the complete suppression in culturability afterward was achieved in approximately twice the time in most of the mentioned studies. The endurance of cells (especially in biofilm form) toward antimicrobials, including NTP exposure, should not be underestimated, and the fact that their viability decreased due to treatment does not guarantee the complete suppression of cell survival. Zhang et al. (2013) used PAW exposed to NTP for 20 min, which resulted in the total suppression of cell survival after 10 min treatment of cells (a 6 log decrease in CFU/ml). In addition, they observed the effect of storage on the efficacy of PAW and found that, after 24 h storage, the same PAW needed 40 min to achieve complete suppression of cell growth. Similarly, Shen et al. (2016) evaluated the effect of temperature and storage time on the effectivity of PAW on *S. aureus* cells. They studied storage temperatures ranging from −80 to 25°C and storage times from 0 to 30 days. For the storage temperature, −80°C proved to be the most efficient in the conservation of the effect of PAW. Without storage, the stated PAW caused a 5 log reduction in the survival counts, whereas after 30 days of storage at −80°C, such reduction dropped to only 3.7 log. In comparison, analogically stored PAW at 25°C lost its antimicrobial activity almost completely, resulting in a 0.8 log reduction. Although storage of PAW at −80°C was proven to be effective, its immediate use is better for achieving greater antimicrobial effects.

The effectivity of PAW against *S. aureus* growth is also affected by experimental arrangements of NTP exposure. Tian et al. (2015) studied the effect of a plasma microjet used for PAW preparation placed above or beneath the surface of the liquid. PAW prepared by exposure to NTP placed above the liquid surface showed a decreased antimicrobial activity in comparison to PAW prepared by placing the electrode beneath the liquid surface. They also found distinct differences in the electrical conductivity, hydrogen peroxide content (reflecting the creation of ROS), and the pH of both types of PAW. PAW created with electrode under the surface of the liquid showed an increase in almost all parameters in comparison to PAW created with the electrode placed above the surface, indicating the greater effectivity of placement beneath the liquid surface for preparing effective PAW.

Lastly, there are also several studies on the use of plasma-activated solutions such as saline, PBS, and even alginate gels. Oehmigen et al. (2010) studied saline and PBS activated by NTP and found the plasma-activated PBS to be more effective than saline, resulting in total inhibition after 15 min treatment of cells. As mentioned in the section on *E. faecalis*, Ercan et al. (2013, 2014) reported on the antibacterial effect of PAW solution of NAC, methionine, threonine, glucose, cysteine, proline, glycine, glutamine, heparin, and arginine against *S. aureus*. Interesting results were also presented by Poor et al. (2014), reporting plasma-activated alginate wound dressing as a strong antimicrobial agent. Alginate gel is a common emulsifier or gelling agent, which is non-toxic but has no meaningful antimicrobial property. On the other hand, alginate gels exposed for 15 s to NTP completely inhibited 10^7^ CFU/ml of *S. aureus* and *A. baumannii* in planktonic forms within 15 and 30 min, respectively. The 80% inhibition of the viability of biofilm cells was observed after 15 min treatment.

### Klebsiella pneumoniae

*Klebsiella pneumoniae*, formerly called Friedländer’s bacillus or *Bacillus mucosus capsulatus*, is a Gram-negative pathogen belonging to the family Enterobacteriaceae. *Klebsiella* spp. is closely related to the *Enterobacter* genus, from which it is difficult to distinguish. It is globally widespread in both community and hospital settings; it commonly occurs in the human intestine, but also survives well in the environment, which is also a frequent source of infection. Patients on ventilators and catheters or with surgical wounds are prone to infections with *K. pneumoniae*. It causes a spectrum of diseases and accounts for about one-third of all Gram-negative infections, including urinary tract infections, pneumonia (mainly in patients in intensive care units), surgical wound infection, sepsis, meningitis (mainly in newborns), intra-abdominal infection, and infections complicating burns (Anderson et al., 2014; Navon-Venezia et al., 2017). It is the second most frequently encountered pathogen in community-acquired urinary tract infections (Effah et al., 2020). *K. pneumoniae* produces an arsenal of virulence factors that aid them in adhesion and invasion, thus helping in establishing the infection. It can cause invasive infections due to fimbrial adhesins and a thick capsule that acts as an antiphagocytic factor (Ahmed and Alaa, 2016). Additionally, it also secretes siderophores, heat-labile exotoxins, and hemolysins (Clegg and Murphy, 2016). *K. pneumoniae* is ranked as one of the top three resistant pathogens according to the WHO priority list of antibiotic-resistant bacteria. Not only its intrinsic resistance but also its acquired resistance to antibiotics that posed a significant challenge for clinicians. In the last few years, there has been a tremendous increase in the rate of extended-spectrum cephalosporin-resistant *K. pneumoniae* producing extended-spectrum β-lactamases (ESBLs). These strains of *K. pneumoniae* are frequently associated with the spread of ESBLs through horizontal gene transfer (Vading et al., 2018). *K. pneumoniae* has also developed resistance to carbapenems, traditionally used as the drugs of last resort for treating resistant Gram-negative infections, leading to substantial increases in mortality and costs of the healthcare system (Reyes et al., 2019). The emergence of the New Delhi metallo-β-lactamase-1 among *K. pneumoniae* has compounded the problem of medical treatment and poses a serious public health threat (Brinkac et al., 2019). The relationships between *K. pneumoniae* and NTP treatment have been studied and usually presented together with other bacteria, mainly Enterobacteriaceae. We did not find a study dedicated exclusively to this bacterium (Supplementary Table 3).

#### Planktonic Form

The sensitivity of planktonic cells to NTP is similar to that of other species. Treatment of suspension cells was described in Gabriel et al. (2016), where a 5 log decrease was observed after 25 min of exposure. The efficiency of decontamination of agar plates was studied in several papers (Justan et al., 2014; Metelmann et al., 2018; Gorbunova, 2019; Lee et al., 2019), where inhibition zones were observed from several seconds to 10 min of exposure depending on the NTP source and arrangement. In addition, a more than 3 log decrease of viable cells on inoculated steel plates was observed after 20 min NTP exposure in the study by Lis et al. (2018).

#### Biofilm

Gilmore et al. (2018) reviewed the fundamental descriptions of biofilms, their specific characteristics, and the mechanisms of their interactions with NTP. In this general overview, *K. pneumoniae* was only briefly mentioned. Furthermore, Gupta and Ayan (2019) reviewed recent advances in NTP application on biofilms. The decontamination and sterilization of numerous bacterial biofilms are summarized here. For *K. pneumoniae*, the use for the sterilization of venous and urinary catheters is proposed. O’Connor et al. (2014) reported on the possibility of using NTP to prevent nosocomial bacterial and viral infections. Methodically, it was proposed to use DBD and plasma jet for the decontamination of skin, wounds, tissue cultures, and medical and dental instruments, including biofilm removal. Good efficacy was reported for several bacteria and viruses. *K. pneumoniae* was mentioned here as an important target, but without specific results. Lee et al. (2019) investigated the antibacterial properties of titanium used for dental implants treated with NTP. *Klebsiella oxytoca* and *K. pneumoniae* were used as the tested species, together with *Streptococcus mutans* and *S. aureus*. The adhesion and the biofilm formation rate of bacteria were significantly reduced on the plasma-treated titanium surfaces compared with those of the untreated samples. Both adhesion and the biofilm formation rate were significantly lowered for Gram-negative bacteria than for the Gram-positive ones. Thus, NTP treatment could be useful for preventing bacterial adhesion and biofilm formation on titanium dental implants.

#### Plasma-Activated Water

Plasma-activated water was used for the decontamination of tiger nut samples in Muhammad et al. (2019), where a 4 log decrease (from an initial 7 log) was observed after 15 min of PAW application. The activation of other various solutions (glucose, heparin, and amino acids) by NTP was described in Ercan et al. (2014). The authors declared that the plasma-activated methionine solution exhibited a strong inhibitory activity against not only *K. pneumoniae* but also other tested bacterial species. In addition, this solution prevented the formation of biofilms by about 70% compared to untreated controls and inhibited the biofilm formation in less than 30 min exposure to the plasma-activated methionine solution. This suggests that plasma-activated solutions have the potential to prevent biofilm formation.

#### Other Applications

As described in detail by El-Sayed et al. (2015), NTP may be used for the treatment of wastewater. This application has already been mentioned in the historical work on plasma production (Siemens, 1857). The design of the ozone generator, known today as an ozonizer, was described there. Wastewater samples were collected from a food processing and a leather processing plant. Twenty-two bacterial species were identified using 16S rDNA sequences. The samples were exposed to NTP for 30–90 s, and extensive results were summarized. *Klebsiella* spp. showed good sensitivity to NTP treatment. From the complex results, the following might be mentioned as typical examples: dominant bacterial groups in leather processing wastewater changed greatly upon exposure to NTP for 30 and 60 s, with *Klebsiella* spp., *Enterobacter aerogenes*, and *Acidithiobacillus ferrooxidans*. Extension of the exposure time to 90 s resulted in an 80% reduction in bacterial populations and the elimination of all bacterial groups, except for the resistant *Pseudomonas* spp. and *Citrobacter freundii*.

Gorbunova (2019) described the possibilities of using NTP in the food industry for processing and preserving meat products. A collection of bacterial strains was exposed in model experiments, where *K. pneumoniae* was inhibited after a 10-min exposure by 36%. Among the other results of this work are the interesting attempts to decontaminate real meat samples, reducing almost half the incidence of contaminating bacteria while maintaining the sensory properties of the exposed meat. Another work (Kan, 2014) described the use of NTP treatment in the textile industry. Besides various aspects of fiber modifications or coloring, the bacterial inactivation and the antimicrobial functionality of textiles were also mentioned. For *K. pneumoniae* inhibition in wool, the combination of enzyme, peroxide, and argon plasma pretreatment is recommended as the most effective.

Dong et al. (2010) described the use of NTP to generate a stable strain of *K. pneumoniae* with improved 1,3-propanediol production. This compound is important due to its large potential in commercial applications, particularly as a monomer of polyesters, polyethers, or polyurethanes. It may be prepared from glycerol by the specific activities of glycerol dehydrogenase, glycerol dehydratase, and 1,3-propanediol oxidoreductase of *K. pneumoniae*. The change of their activities gave rise to the improved 1,3-propanediol production, which could be considered as a mutation to a new strain designed as Kp-M2.

### Acinetobacter baumannii

*Acinetobacter baumannii* is recognized as an important opportunistic nosocomial pathogen, most often encountered in the hospital environment, particularly in intensive care units and surgical ward (Eveillard et al., 2013). It causes a spectrum of infections that includes skin and soft tissue infection, pneumonia, bacteremia, secondary meningitis, and urinary tract infection (Dexter et al., 2015). As compared to other Gram-negative pathogens, *A. baumannii* is known for its environmental endurance and for being viable for up to 5 months on inanimate surfaces (Kramer et al., 2006). As it can withstand dry conditions for extended periods, it is most commonly isolated from reusable medical equipment (Almasaudi, 2018). The outer membrane protein (Omp A), which acts as a cytotoxin, is one of the important virulence factors that induce apoptosis in epithelial cells, which leads to its early colonization (Choi et al., 2005). Biofilm formation in *A. baumannii* facilitates its attachment to abiotic and biotic surfaces, including those of medical devices and host tissues. The growth of *A. baumannii* in unfavorable conditions is due to its ability to form biofilms. The phospholipases (phospholipase C and phospholipase D) produced are involved in serum resistance (Jacobs et al., 2010). The persistence of *A. baumannii* within epithelial cells is dependent on the ability of bacteria to synthesize and transport the produced siderophore (Hasan et al., 2015). Twitching motility allows them to spread rapidly on semisolid and certain abiotic surfaces (Harding et al., 2013). With a restricted number of virulence factors, which are not always present in all strains of *A. baumannii*, the mechanism behind their success is of interest to researchers (Morris et al., 2019). Due to its ability to acquire antibiotic resistance, *A. baumannii* is considered to be one of the most successful pathogens in the healthcare setting. Acquisition of foreign determinants and the upregulation of innate resistance make it an important multidrug-resistant bacterium worldwide. Drug resistance in this bacterium is due to mechanisms such as the modification of the target site, production of β-lactamases, efflux pumps, permeability defects, and aminoglycoside-modifying enzymes (Touchon et al., 2014; De Silva and Kumar, 2019). *A. baumannii* strains carrying *bla*_IMP_-encoded imipenem metallo-β-lactamases and *bla*_OXA_-encoded oxacillinase serine-β-lactamase showing resistance to colistin and imipenem have emerged, and they are resistant to almost all known antibiotics available to clinicians. According to its clinical significance, *A. baumannii* is classified as a WHO priority 1 pathogen.

#### Planktonic Form

The initial series of references is devoted to the ability of NTP to inactivate *A. baumannii* inoculated on the surface. The following papers focused on wet surfaces. Svarnas et al. (2019) reported that 1 ml of PBS suspension of 3 × 10^6^ CFU/ml was inactivated after 20 min of treatment. On agar surface inoculated with 10^4^ CFU/cm^2^, NTP exposure of 240 s created a circular inhibition zone of 15 mm in diameter. Atta (2019) exposed cultures on agar surfaces and obtained circular inhibition zones of 4 cm in diameter after 60 s of NTP exposure. Moreover, damage of the DNA, protein structure, and bacterial morphology caused by the NTP exposure was also mentioned. In Kolb et al. (2012), *A. baumannii* was inactivated on agar surface at 10^5^ CFU/cm^2^ concentration. Complete inhibition on a 2 × 2 cm^2^ area was observed within 120 s. Heller et al. (2012) used NTP to inactivate *A. baumannii* on the surface of both agar plates and porcine skin, which mimics clinical application. The 6 log decrease in bacterial count meant an almost complete inactivation on agar plates, which was observed after 215 s of NTP exposure. On porcine skin, a reduction of only 2–3 log was observed. This lower effect may probably be explained by the scabrous surface serving as protection for the bacteria.

Cahill et al. (2014) inactivated MRSA, VRE, and *A. baumannii* on dry surfaces. Contaminated surfaces of marmoleum, mattress, polypropylene, powder-coated mild steel, and stainless steel were exposed to NTP for up to 90 s. The exposure successfully reduced the bacterial load by 1.7 log for *A. baumannii*. A similar multi-jet arrangement applied on the same bacteria was presented in Cahill et al. (2017). NTP was applied to 5-cm^2^ sections of stainless steel and mattress for up to 45 s, where at least 3–4 log and 3–6 log reductions were achieved on the mattress and stainless steel, respectively. Lis et al. (2018) inoculated *A. baumannii*, *E. faecium*, *S. aureus*, and *K. pneumoniae* on a stainless steel surface. They reported a 3 log decrease after 20 min of NTP exposure. However, after the addition of BSA serving as protection for the bacteria, the inactivation efficacy fell to only a 2 log decrease.

The last two papers described NTP decontamination of various types of suspensions. Parkey et al. (2015) used helium and helium/air mixture (97:3) gas NTP to inactivate *A. baumannii* and *S. aureus* in 150 μl of PBS suspension. While the pure helium gas NTP was only less effective, the helium/air mixture gas NTP led to the full inactivation of *A. baumannii* (7 log decrease). A complete inactivation of *A. baumannii* was also reported by Ruan et al. (2018), who used the NTP in open air. A rapid decrease of the surviving bacteria was observed in 2 ml suspension of 10^6^ CFU/ml concentration after 5 min of NTP exposure; a total inactivation occurred after 10 min.

#### Biofilm

Flynn et al. (2019) used the helium/oxygen plasma jet for up to 540 s to inactivate the *A. baumannii* biofilm grown for up to 72 h. As expected, the younger 24-h-grown biofilm was more sensitive to the NTP treatment than were the biofilms grown for 48 and 72 h. While the 24-h biofilm was inactivated from the initial 10^6^ CFU/peg (i.e., peg lid of the Calgary Biofilm Device) to approx. 10 CFU/peg in 540 s, the others were inactivated to only 10^2^–10^3^ CFU/peg. The other differences presented in this paper were not statistically significant. Lis et al. (2018) reported a 4 log reduction of the biofilm of carbapenem-resistant *A. baumannii* isolate formed on stainless steel after a 20-min exposure to NTP.

#### Plasma-Activated Water

As also mentioned under *E. faecalis*, Ercan et al. (2013, 2014) reported the antibacterial effect of plasma-activated solutions of NAC, methionine, threonine, glucose, cysteine, proline, glycine, glutamine, heparin, and arginine against *A. baumannii*. Also, as mentioned under *S. aureus*, Poor et al. (2014) reported that a plasma-activated alginate wound dressing completely inhibited within 15 min 10^7^ CFU/ml of *A. baumannii* in planktonic form.

### Pseudomonas aeruginosa

*Pseudomonas aeruginosa*, a notorious opportunistic nosocomial pathogen, is the principal cause of morbidity and mortality among immunocompromised individuals. It gains entry and establishes itself in high-risk patients, such as those in intensive care with cystic fibrosis and with immune suppression causing life-threatening infections (Fazeli and Momtaz, 2014). Infection outbreaks occur due to the colonization of this bacterium on the surface of medical devices such as ventilators, catheters, and bronchoscopes (Mulcahy et al., 2014). The metabolic versatility of *P. aeruginosa* permits its survival in extreme environments and hospital outbreaks, where it encounters different routinely used disinfecting agents and soaps (Dantas et al., 2008). *P. aeruginosa* is implicated in causing several infections such as pneumonia, wound infections including secondary infection of burns, urinary tract infection, and bacteremia (Ranjan et al., 2017). This Gram-negative bacterium is one of the highly successful pathogens as it is armed with a wide range of virulence factors that include structural motives, extracellular substances, and biofilms (Balasubramanian et al., 2013). *P. aeruginosa* is equipped with a sophisticated resistance mechanism that provides it with intrinsic resistance against various antibiotics. This natural resistance is due to the presence of various transport systems and the low permeability of the outer membrane (Ali et al., 2018). A thick extracellular matrix of biofilms is also a key factor of drug resistance as it prevents drug penetration of the cell wall (Yan and Wu, 2019). Furthermore, the pathogen frequently acquires resistance genes through horizontal gene transfer (Partridge et al., 2018). The emergence of multidrug-resistant and extensively drug-resistant *P. aeruginosa* globally limits the therapeutic options. The most common mechanisms adopted by the strains of *P. aeruginosa* are target site modifications, alterations in porin channels, presence of extended-spectrum β-lactamases, and efflux pumps (Eichenberger and Thaden, 2019). Carbapenem resistance is mostly due to chromosomally mediated AmpC production combined with porin change or the presence of *K. pneumoniae* carbapenemases (KPC) and β-lactamases encoded by *bla*_VIM_ or imipenem β-lactamases (Santajit and Indrawattana, 2016). The WHO Report of 2017 considered *P. aeruginosa* as priority 1 pathogen and addressed the need for developing new antimicrobials to combat the resistance of this pathogen.

#### Planktonic Form

The efficiency of NTP against *P. aeruginosa* in planktonic form was examined in many relevant studies (Gadri et al., 2000; Ermolaeva et al., 2011; Alkawareek et al., 2012a; Lunov et al., 2016b; Mai-Prochnow et al., 2016; Mohd Nasir et al., 2016; Abbas et al., 2017; Navon-Venezia et al., 2017; Kondeti et al., 2018; Mohammed and Abbas, 2018; Humud, 2019). For details, see Supplementary Table 5 (also with other studies referred to in this section). Alkawareek et al. (2014) tested a selected panel of clinically significant bacterial species that included *Bacillus cereus*, MRSA, *E. coli*, and *P. aeruginosa* exposed in planktonic form directly to NTP. All these bacteria were completely inactivated within 2 min of NTP exposure. The reduction of the cell population was 10^7^ CFU/ml in the case of *P. aeruginosa*. In addition, the damaging effects of NTP on the cellular components, including DNA, a model protein enzyme, and lipid membrane integrity and permeability, were observed in this study. The 10-min NTP exposure was used for the investigation of its impact on the lipopolysaccharide toxicity of *P. aeruginosa* in Barakat et al. (2019), where a complete reduction in endotoxin concentration was achieved.

In Puač et al. (2014), bacterial suspensions of *P. aeruginosa* and *E. faecalis* were treated for 60–180 s. Complete inhibition of bacteria was achieved after 180 s of NTP exposure (10^3^ CFU/ml decrease). A more efficient sterilization was achieved in the case of *P. aeruginosa*. Lunov et al. (2016a) treated *E. coli*, *P. aeruginosa*, *S. aureus*, and *Bacillus subtilis* inoculated on agar plates. After 60 s of NTP exposure, no survival was detected. The SEM images confirmed damage of the cells. Sohbatzadeh et al. (2010) also treated bacterial suspension of *P. aeruginosa* (tested in comparison with *B. cereus* and *E. coli*) with 10 min of NTP exposure and observed total inactivation of cells determined as a decrease in optical density. In the study of Yang et al. (2009), *P. aeruginosa* on contaminated polyethylene terephthalate (PET) sheets was treated using NTP. The results, confirmed by SEM, showed total inactivation in a short time (60 s). In Nishime et al. (2017), NTP was applied directly on agar plates inoculated by *E. faecalis*, *P. aeruginosa*, and *Candida albicans*. For *P. aeruginosa*, an inhibition zone of 100 mm^2^ was detected after 180 s of NTP exposure.

Liu et al. (2021) studied the action of NTP on *A. baumannii* exposed on the surface of Petri dishes. NTP was produced by corona discharge on an array of pin electrodes. The positive discharge appeared to be more effective than the negative discharge. A 99.99% sterilization efficiency was achieved within 9 min.

#### Biofilm

*Pseudomonas aeruginosa* was examined by Alkawareek et al. (2012a) alongside other microorganisms (*B. cereus*, *S. aureus*, and *E. coli*) for the eradication of its biofilm pre-formed on the peg lid of the Calgary Biofilm Device. An exposure time lower than 240 s was necessary for the complete eradication of Gram-positive bacterial biofilms, while the Gram-negative biofilms required a longer treatment time (6 log decrease after 600 s). The following research by the same laboratory group (Alkawareek et al., 2012b) confirmed the eradication of *P. aeruginosa* biofilm pre-formed on the peg lid of the Calgary Biofilm Device and polycarbonate coupons after direct exposure to NTP. The authors demonstrated that the parameters of NTP generation (particularly the frequency) had a significant effect on the bacterial inactivation rate. There was total decrease in the number of surviving cells (6.2 log CFU/ml). The anti-biofilm activity of NTP against *P. aeruginosa* was also confirmed using the XTT {sodium 3′-[1-[(phenylamino)-carbony]-3,4-tetrazolium]-bis(4-methoxy-6-nitro)benzene-sulfonic acid hydrate} metabolic assay and CLSM. A similar experimental setup was used by Patenall et al. (2018), who investigated the effect of NTP against *P. aeruginosa* biofilm grown on polycarbonate membranes exposed to NTP at different stages of its development. The results showed that the stage of biofilm formation exposed to NTP has a crucial role in its ability to overcome the NTP effect and regrow. A reduction of the culturability of cells of 10^4^–10^5^ CFU/ml was achieved for the early stage biofilm (0–8 h) exposed to NTP, while only a 10^2^-CFU/ml decrease was shown for the developed and mature biofilms (12–24 h). These findings were confirmed using SEM and CLSM. The study of Mai-Prochnow et al. (2016) described the effect of NTP against different single-species (*B. subtilis*, *Staphylococcus epidermidis*, *Pseudomonas libanensis*, and *P. aeruginosa*) and mixed-species bacterial biofilms formed on glass and stainless steel coupons. The authors reported that the efficacy of NTP is directly correlated with the bacterial cell wall thickness. While the biofilm of *B. subtilis* (55.4-nm cell wall) showed less susceptibility to NTP exposure (<10 CFU/ml reduction after 600 s treatment), *P. aeruginosa* (2.4-nm cell wall) was almost completely eradicated (3.5 log CFU/ml decrease) at the same conditions. In addition, the cell membranes and the biofilm matrix also played significant roles. Mixed-species biofilms corresponded to the properties of the least sensitive species in the culture. In Gupta et al. (2017), the effectivity of NTP exposure on the biofilm of *P. aeruginosa* pre-formed on titanium coupons was also examined in combination with the biocide CHX digluconate, which was used for 5–15 min. The authors found that complete elimination of the biofilm (10^8^ CFU/ml reduction) can be achieved after this combinatorial treatment. When using NTP alone, only a 10^3^-CFU/ml decrease was achieved. Soler-Arango et al. (2019) studied the eradication of *P. aeruginosa* biofilm pre-formed on stainless steel 316L, which showed 6.5 log CFU/ml decrease after 30 min of NTP exposure. Moreover, significant damage on the biofilm matrix was described, where mostly the carbohydrates and environmental DNA (eDNA) succumbed to chemical and structural changes.

In two works from one research group (Zelaya et al., 2010; Vandervoort and Brelles-Mariño, 2014), *P. aeruginosa* biofilm was inactivated. Firstly, a significant reduction of the adhesiveness of *P. aeruginosa* biofilm to borosilicate coupons in batch cultures and a reduction of the thickness of the grown biofilms were reported. In addition, after 5 min of NTP exposure, no culturable cells of *P. aeruginosa* were detected. The group’s further research in a continuous culture system showed total decrease (8 log CFU/ml) of culturable cells after 30 min of NTP exposure.

Triandafillu (2003) investigated *P. aeruginosa* adhesion on PVC surfaces previously exposed to oxygen–plasma for 120 s. This treatment made a hydrophilic surface of PVC, therefore reducing the number of adhering bacteria by as much as 70% (adhered cells counted using a light microscope). However, it was considered as insufficient for the prevention of *P. aeruginosa* colonization of PVC endotracheal intubation devices. Gabriel et al. (2016) applied a cocktail of four *P. aeruginosa* strains on the surface of stainless steel 316 and 304 for cell attachment. NTP was used for the early stage biofilm treatment for 90 s, with a 99.9% reduction in the cell population (a decrease of 5 log CFU/ml was achieved). In our previous study (Paldrychová et al., 2019), we examined the effect of NTP on the redevelopment of pre-formed biofilms of different *P. aeruginosa* strains on Ti-6Al-4V titanium alloy. There was a significant difference in the sensitivity of non-hospital and clinical strains to NTP action. While the biofilms of the clinical isolates were not eradicated after 60 min of NTP exposure, the non-hospital strains were almost eliminated. In addition, quorum sensing signaling molecules [acyl-homoserine lactones (AHL)] were detected and the lower level of AHL production was determined in non-hospital strains. In Flynn et al. (2016), commercially available AHL molecules (produced usually by *P. aeruginosa*) were exposed to NTP for 240 s. This treatment caused the degradation of the AHL molecules and their conversion into a series of by-products. In Ziuzina et al. (2014), the biofilm of *P. aeruginosa* grown on 96-well microplates and glass coverslips was exposed to NTP for 300 s. This treatment reduced the metabolic activity of biofilm cells by 70%, determined using the XTT assay. This research was expanded in Ziuzina et al. (2015) by monitoring the efficiency of the discharge burns under the same conditions on the reduction of quorum sensing-regulated virulence factors of *P. aeruginosa*. The production of pyocyanin was significantly inhibited after a short treatment time (60 s), but the reduction of elastase LasB was notable only after 300 s and no reduction in the actual biofilm formation was achieved according to the CFU counts.

Hübner et al. (2010) compared the impact of NTP exposure for 30 s with the action of CHX and polyhexanide (PHMB) on the biofilm of *P. aeruginosa* grown on polystyrene microplates and silicone swatches. A 10^3^-CFU/ml decrease was achieved in *P. aeruginosa* biofilm exposed to NTP, while PHMB showed a reduction of 10–100 CFU/ml. The most effective was CHX applied on the biofilm formed on silicone swatches (10^4^ CFU/ml decrease). In Matthes et al. (2013b), the same research group investigated the anti-biofilm activity of NTP and CHX against *P. aeruginosa* and *S. epidermidis* biofilms grown on 96-well microplates. The effectiveness of both agents was comparable, but NTP treatment led to a higher reduction effect (5.5 log CFU/ml decrease) depending on the exposure time and the gas used. Moreover, Matthes et al. (2013a, 2014) revealed very similar results on polycarbonate disks. NTP pretreatment was used for the enhancement of the action of antibiotics (gentamicin, ceftazidime, and polymyxin B) against *P. aeruginosa* biofilm in our recent study (Paldrychová et al., 2020). The strongest anti-biofilm effect was observed in the case of the combined action of NTP and gentamicin. The *P. aeruginosa* biofilm was completely eradicated by these agents, which was also confirmed using fluorescent microscopy and SEM.

#### Plasma-Activated Water

As mentioned under *E. faecalis*, Ercan et al. (2013) reported that a total inactivation of 10^7^ CFU/ml of *P. aeruginosa* in both planktonic and biofilm forms was observed after 15 min with the plasma-activated solution of NAC.

#### Other Applications

A special application of NTP was examined by Scally et al. (2018), who described the possibility of treating low-density polyethylene for the acceleration of the biodegradation of this material with *P. aeruginosa*. NTP was applied on the surface of polyethylene for 300 s. The biodegradation of this material was increased, probably due to an increase in oxidative species causing better cell adhesion and acceptance on the polymer sample surface. Another special application of NTP was investigated by Hammann et al. (2010) when testing eyeballs extracted from commercially slaughtered pigs artificially contaminated with *S. aureus* and *P. aeruginosa*. The efficacy of NTP exposure for 600 s resulted in a higher effectivity (RF = 2.4–2.9) compared with that of povidone iodine and polyhexanide. An *in vivo* study was also described by Yu et al. (2011), who investigated the effect of NTP on *P. aeruginosa* colonization on skin wound in mice. The counts of the bacterial colonies decreased from an initial 9 log to 3 log CFU/ml; therefore, it was suggested that NTP facilitates wound healing by suppressing bacterial colonization.

### *Enterobacter* spp.

*Enterobacter* spp., a member of the family Enterobacteriaceae, is a genus of Gram-negative bacteria that has many features in common with the genus *Klebsiella*, but is readily distinguished by their motility. The significant species are mainly *E. aerogenes* (synonymous with *Klebsiella aerogenes*) and *E. cloacae*. The *Enterobacter agglomerans* species has been transferred to the genus *Pantoea*. The normal habitats of *Enterobacter* spp. are soil and water, but they can also be found as a common constituent of the human intestine microflora as commensal microorganisms. On the other hand, they have been reported to cause hospital-acquired infections particularly of the urinary or lower respiratory tract; in addition, they are also known to cause bloodstream infections (Mezzatesta et al., 2012). *Enterobacter sakazakii* also causes meningitis in children. They are primarily resistant to a number of antibiotics, including penicillins, first- and second-generation cephalosporins, and amoxicillin/clavulanic acid, owing to the production of chromosomal AmpC β-lactamases. Moreover, under selective pressure, they also become resistant to third-generation cephalosporins and monobactams (Hilty et al., 2013). The emergence of *Enterobacter* spp. strains capable of the production of a wide spectrum of β-lactamases and carbapenemases, such as the Verona integron-encoded metallo-β-lactamases and oxacillinase serine β-lactamases, has caused significant health concerns (Castanheira et al., 2011). Carbapenem-resistant *Enterobacter* spp. were included in the critical priority list of pathogens published by World Health Organization (2017).

Articles on the inactivation of *Enterobacter* spp. by NTP are rather rare. In this regard, the bacterium differs little in its susceptibility toward NTP from other representatives of Enterobacteriaceae.

#### Planktonic Form

The possibility of the inactivation of microorganisms on the surface of polyethylene and polyvinyl chloride substrates using NTP is mentioned in Tiwari and Chaturvedi (2018). For *Enterobacter* spp., a short exposure time of 1 min was required for their inactivation. For other bacteria, e.g., *Listeria* or *Klebsiella* spp., an exposure time of at least 3 min was required (Agnihotri et al., 2018). On the other hand, in the small number of studies on such problems, 3 min was proven to be a decent treatment time, as shown, for example, in Parkey et al. (2015). They achieved complete suppression of the planktonic growth of *E. cloacae* on polystyrene microtiter plates after NTP treatment for 3 min. A high antimicrobial effect of NTP was also proven in the case of *E. agglomerans* in Ren et al. (2012). They achieved a 90% decrease of the viability of planktonic cells with a 4-min treatment. Similarly, total inhibition of *E. aerogenes* growth on stainless steel (7 log CFU/ml decrease) after a 3-min exposure to NTP was achieved in Lu et al. (2011).

#### Biofilms

Although this species is not often linked to its biofilm formation and to its further virulent potential, there are a handful of studies on the ability of NTP to inhibit the biofilm formation of the genus *Enterobacter*. Mai-Prochnow et al. (2016) tested the efficacy of NTP exposure on *E. cloacae* biofilms formed on stainless steel and found that treatment for 10 min caused a 3.5 log CFU/ml reduction in survival counts (approximately by half, original inoculum of 6 log CFU/ml). The anti-biofilm effect of NTP on *E. cloacae* was further observed only by the previously mentioned study of Flynn et al. (2015), who investigated NTP treatment of all the ESKAPE pathogens. In this study, total inhibition of biofilm formation on microtiter plates with NTP treatment for only 2 min was achieved. It is interesting to compare the small amount of data on the susceptibility of *E. cloacae* planktonic cells to NTP with those of biofilm cells, as the average effective treatment time for NTP to achieve complete inhibition of planktonic growth is 3 min, but Flynn et al. (2015) completely suppressed the biofilm formation of this species after only 2 min. This was highly dependent on the arrangement of the device for NTP generation, which also generally results from the parameters described in Supplementary Table 6.

#### Other Applications

In clinical settings, a multiple-cycle NTP exposure was proven effective in the treatment of heart assist device-related infections. Urayama and McGovern (2018) described the NTP treatment of patients with infected left ventricular assist device (LVAD) (applied to cure heart failure). The infection was located in the pump pocket or on the driveline. Out of six treated patients, *Enterobacter* spp. were found in three, usually accompanied by *Pseudomonas* spp., *Klebsiella* spp., or *Staphylococcus* spp. Treatment for 2–9 weeks caused complete or nearly complete healing, which strongly highlights the great medical potential of NTP.

El-Sayed et al. (2015) described the efficacy of NTP in the decontamination of wastewater containing *E. aerogenes*. The presence of the species, among others, was completely suppressed in wastewater samples treated with NTP for 30 s.

Finally, NTP treatment may be a useful tool for mutation. In addition to the information mentioned above, Ren et al. (2012) carried out an efficient agar plate mutagenesis and screening technique for improving the mutation frequency. *E. agglomerans* is well known as a phosphate-solubilizing plant-associated bacterium, and NTP was applied to its mutation in order to improve the phosphate-solubilizing activity. The results showed that the phosphate-solubilizing activity of mutants increased compared with that of the original strain, and the phosphate-solubilizing activity of the best mutants was 1.49-fold that of the original strain. It demonstrated that NTP treatment has high efficiency and that it will be a useful method for mutation. Comparable results were presented by Lu et al. (2011): the positive mutant of *E. aerogenes* obtained after 3 min of NTP exposure showed a 26% increase of the total hydrogen yield per mole of glucose and was genetically stable after more than 25 subcultures. The increase of the hydrogen production by the mutant strain makes it promising to produce hydrogen as an alternative clean energy source.

## Conclusion

Non-thermal plasma has already found extensive use in various fields of industry, in food processing and packaging and in others. It is also readily used in medicine for the actual treatment of patients, namely, for wound healing, in antitumor therapy, in dental medicine and dermatology, for the treatment of diabetic ulcers and chronic infections of colonized implants, and for the treatment of fungal diseases. This holds also for the surface disinfection of medical materials and devices such as sutures and respirators or various liquids. This review demonstrates the importance of NTP as an effective antimicrobial tool for the inactivation of prominent pathogens of the ESKAPE group. The most important advantage of NTP as an antimicrobial tool is, among others, the fact that, unlike antibiotics, it eliminates the possibility of the development of resistance, which is relevant especially for the poly-resistant *S. aureus* and *P. aeruginosa*. This review shows that diverse experimental setups have already been used to inactivate ESKAPE, i.e., different plasma sources operating at different electrical parameters, different auxiliary gases, etc. It is also clear that the inactivation efficiency differs for different configurations. The inactivation effectiveness also depends on the nature of the exposed bacteria: in general, Gram-positive bacteria appear to be less susceptible toward NTP treatment compared with Gram-negative bacteria. Such lower sensitivity is probably due to the thicker layer of peptidoglycan in the cell wall of Gram-positive bacteria, which acts as a shield against the reactive particles in NTP.

## Author Contributions

VS and EV conceptualized the study. VS, EV, PK, and JJ reviewed and edited the manuscript. EV and JJ supervised the study. All authors wrote the original draft and have read and approved the submitted manuscript.

## Conflict of Interest

The authors declare that the research was conducted in the absence of any commercial or financial relationships that could be construed as a potential conflict of interest.

## Publisher’s Note

All claims expressed in this article are solely those of the authors and do not necessarily represent those of their affiliated organizations, or those of the publisher, the editors and the reviewers. Any product that may be evaluated in this article, or claim that may be made by its manufacturer, is not guaranteed or endorsed by the publisher.
