# Supplementary material for: Non-thermal Plasma Treatment of ESKAPE Pathogens: A Review
Source: Front Microbiol. 2021 Oct 12;12:737635. doi: 10.3389/fmicb.2021.737635 (PMC8546340; doi:10.3389/fmicb.2021.737635)
Supplement: Supplementary file 1 [file Data_Sheet_1.PDF]

Supplementary Table 1: Overview of NTP inactivation of *Enterococcus* spp. at various experimental parameters

Microorganism	NTP source				Treated sample (inoculum)	Results		Reference
	Discharge	Voltage	Frequency	Gas (flow rate)		Treatment time	Cell inactivation (CFU)	
E. faecium vancomycin resistant isolate	circular plasma system, SMD	10 kV	2 kHz	air (5 L/min)	suspension on stainless steel plates (6 log)	20 min	3.66 log	(Lis et al., 2018)
E. faecium CCM (number unspecified)	corona discharge	10 kV	*	air (5 L/min)	suspension on agar plates (6 log)	16 min	inhibition zone 38 mm^2	(Scholtz et al., 2010)
E. faecalis ATCC 29212	plasma jet, DBD type (AC)	*	*	helium (2 or 4 L/min)	bacteria on agar plates	3 min	inhibition zone 225 mm^2	(Nishime et al., 2017)
E. faecium clinical isolate	microwave torch (MicroPlaSter β device)	18 V DC	2.45 GHz	argon	suspension on agar plates (3-5 log)	10 min	total	(Ermolaeva et al., 2011)
E. faecalis unspecified	plasma brush, AC	22 kV	11kHz	helium/:oxygen (0-10 %) (4.5 L/min)	suspension on sterile filter papers (7 log)	1 min	total	(Chen et al., 2012)
E. faecium ATCC 6057	plasma jet	1-5 kV	1.5 MHz	argon (8 L/min)	suspension on agar plates (2.5 log)	three times 2 min	1.9 log	(Daeschlein et al., 2010)
E. faecium and *E. faecalis* clinical isolates	plasma jet	1-5 kV	1.5 MHz	argon (8 L/min)	suspension on agar plates	3 s	inhibition zone 10-20 mm^2	(Daeschlein et al., 2014)
	DBD		0.1-0.4 kHz	air		3 s	285-479 mm^2	
E. faecalis ATCC 29212	plasma jet	8 kV	10 kHz	helium:oxygen (2 %) (1 L/min)	suspension on agar plates, biofilm on nitrate membrane filters (6 log)	15 min	inhibition zone 8.2 cm (diam.); total biofilm	(Cao et al., 2011)
E. faecium ATCC 6057	SMD plasma jet	10 kV	1 kHz	air	suspension on stainless steel carrier	10 min	2 log	(Klämpfl et al., 2014)

					(8 log)			
E. faecium DSM 25390	plasma jet	6 kV	20 kHz	oxygen (0.5 %) + helium (99.5 %) (2 L/min)	suspension cells (8 log)<br>biofilm in microtiter plate (5 log)	120 s<br>240 s	total<br>total	(Flynn et al., 2015)
E. faecalis ATCC 29212	teflon tube jet	18 kV	10 kHz	argon:oxygen (2 %) (5 L/min)	biofilm in root canals (8.12 log)	10 min	total	(Pan et al., 2013)
E. faecalis ATCC 29212	DBD plasma jet	16 kV	10 kHz	helium:oxygen (5 L/min)	biofilm in root canals (8.5 log)	5 min	total in middle parts of the roots	(Üreyen Kaya et al., 2014)
E. faecalis ATCC 29212	kINPen08	*	*	argon:oxygen (1 %) (5 L/min)	biofilm in root canals (6 log)	12 min	2 log	(Hüfner et al., 2017)
E. faecalis ATCC 29212	plasma dental probe	6 kV	1 kHz	argon:oxygen (1 %) (1 L/min)	biofilm on hydroxyapatite discs (7 log)	5 min	1.2 log	(Jiang et al., 2012)
E. faecalis ATCC 29212	plasma jet	8 kV	8 kHz	helium:oxygen (1:0.01 L/min)	biofilm on cover slips or in root canals (7 log)	15 min	6 log	(Du et al., 2012)
E. faecalis ATCC 29212	plasma jet	8 kV	8 kHz	helium:oxygen (1:0.01 L/min)	biofilm on sterile bovine dentin discs (7 log)	2 min	50% of dead cell volume	(Du et al., 2013)
E. faecalis ATCC 29212	plasma jet	8 kV	8 kHz	helium flowing through 3% hydrogen peroxide (2 L/min)	biofilm in root canals (8 log)	4 min	7 log	(Zhou et al., 2016)
E. faecalis ATCC 29212	quartz tube jet	18 kV	10 kHz	argon:oxygen (2 %) (5 L/min)	biofilm in root canals (8 log)	12 min	total	(Li et al., 2015)
E. faecalis DSM 2570, ATCC 2921	SMD	3.5 kV	4 kHz	air	biofilm in petri dishes (untreated control 14.3 log)	10 min	5 log	(Theinkom et al., 2019)
E. faecalis NCTC 775	SBD	*	*	air	mixed spp. biofilms on PVC coupons (6.3 log)	240 s	4 log	(Modic et al., 2017)
E. faecalis unspecified	DBD	30 kV	1.5 kHz	air	plasma activated NAC for treatment of cell suspension or biofilm (7 log)	15 min (3 min activated NAC)	total	(Ercan et al., 2013)
E. faecalis ATCC 29212	DBD	31.4 kV	1.5 kHz	air	plasma activated aminoacid (methionine, threonine, cysteine) solutions for treatment of biofilms	activated solutions for 2 min (15 min application)	more than 50 %	(Ercan et al., 2014)

AC – alternating current, CFU – colony forming units, DBD – dielectric barrier discharge, NAC – N-acetylcysteine, SMD – surface microdischarge, * – unspecified parameter

Supplementary Table 2: Overview of NTP inactivation of *Staphylococcus aureus* (*S.a.*) strains at various experimental parameters

Microorganism	NTP source				Treated sample (inoculum)	Results		Reference
	Discharge	Voltage	Frequency	Gas (flow rate)		Treatment time	Cell inactivation (CFU)	
S.a. ATCC 25923	afterglow corona	20 kV DC	58 kHz	air (2.5 m/s)	suspension on glass slides (8.5 log)	24 h	2.5 log	(Mok et al., 2015)
S.a. NCTC 8325	corona	10 kV DC	*	air	4 mL suspension on agar plate (7 log)	20 min	5.35 log	(Xu et al., 2018)
S.a. (squid contaminant)	corona plasma jet	20 kV DC	58 kHz	air (2.5 m/s)	squid shreds	3 min	0.9 log	(Choi et al., 2017)
S.a. unspecified	commercial device LK/JKFY100	*	*	*	disinfection of air in hospitals	6 min	total	(Wang et al., 2019)
S.a. ATCC 25923	commercial device CUTE series	*	*	*	microdrops of suspension spread on	5 min	4 log (99.99 %)	(Lee et al., 2015)
					food packaging materials	10 min	total	
S.a. NRS193	DBD	120 V AC	0.06 kHz	humid air	suspension on agar plate (8 log)	10 min	4 log	(Burts et al., 2009)
S.a. ATCC 25923	DBD	14 kV DC	10 kHz	air	non-adapted cells	30 s	7.1 log	(Liao et al., 2018a)
					acidity adapted after 24 h (log 8)	30 s	7.5 log	
					heat, osmosis etc. adapted after 24 h	30 s	3 log and less	
S.a. ATCC 25923	DBD	14 kV DC	10 kHz	air	30 ml of sample solution in petri dish (6 log)	10 min	2.55 log	(Liao et al., 2018b)
					combination with ultrasound	5 min + 20 min US	total	
S.a. KCTC 11764	DBD	3.8 kV	4 kHz	air	suspension on pork jerky pieces blended and plated on agar (6 log)	30 min	1 log	(Yong et al., 2019)
S.a. ATCC 6538	DBD	85 kV	*	air	suspension on solid surface (8 log)	60 s	2.3 log	(Huang et al., 2018)
S.a. ATCC 16200/MRSA KCCM 40510/PRSA KCCM40512/GRSA	DBD	4.5 kV	*	humid (20-50%) air flow speed 4.5 m/s	suspension on glass strips (6.5 log)	60 min (low humidity; ATCC strain)	1.5 log	(Ki et al., 2019)

KCCM11812				humid (70-90 %) air flow speed 4.5 m/s		60 min (high humidity; ATCC strain/MRSA/PRSA/GRSA)	total/3 log/5 log/3 log	
S.a. ATCC 25923/ATCC 6538	DBD	4-10 kV	20-60 kHz	nitrogen:oxygen (100:1) (60; 600 cc/min)	paprika powder loaded with drops of suspension (8 log)	120 s (1 kW) (5 cycles of treatment)	1.8 log	(Choi et al., 2018)
					combination with radiofrequency heating (RFH)	120 s (1 kW) + 120 s RFH (1.5 kW) (2 cycles of treatment)	total	
S.a. CGMCC 1.2465	DBD plasma jet	*	20 kHz	air (4.33 L/min)	suspension in 60 ml water	7 min	total	(Zhang et al., 2017)
					then plated on agars (6.6 log)	5 min	3.4 log	
S.a. ATCC 6538	DBD plasma jet	2 kV AC	38 kHz	argon flow speed 8.82 m/s	50 µL suspension on petri dish (8 log)	3 min	6.78 log	(Guimin et al., 2009)
S.a. unspecified	DBD plasma jet	0-7.5 kV AC	12 kHz	helium (4 L/min)	dried suspension on agar plates	15 min	inhibition zone diameter 5.3 cm	(Al-rawaf et al., 2018)
S.a. ATCC 12600	DBD plasma jet	6.8 kV DC	1.5 kHz	98-100% argon + 1-2% oxygen (4 L/min)	PAW prepared by two discharges (activated for not described time)	1 h (DBD plasma jet)	0.15-3.06 log	(Royintarat et al., 2019)
	DBD cylindrical	15 kV DC	20 kHz		mixed with cell suspension (8 log)	1 h (DBD cylindrical)	0.03-2.85 log	
S.a. ATCC 6538	DBD plasma jet	5 kV	30 kHz	helium: oxygen (8 L/min)	suspension on agar plates (inoculum unspecified)	3 min	99%	(Deng et al., 2018)
S.a. ATCC 6538	DBD plasma jet	5.8 kV	*	helium	suspension in microtiter plate (7 log)	3 min	0.7 log	(Parkey et al., 2015)
		5.8 kV		helium + 3 % air		3 min	4.8 log	
S.a. ATCC 51811	DBD reactor	0.85 kV	13560 kHz	helium:air (1.5 L/min)	suspension on agar plate (9 log)	2 min	total	(Solís-Pacheco et al., 2017)
S.a. ATCC 6538	microwave torch	18 V DC	2450000 kHz	argon	suspension on agar plate (3-5 log)	5 (10) min	90 (99) %	(Ermolaeva et al., 2011)
S.a. clinical isolate 85						5 min	total	
S.a. clinical isolate 78					rat model of superficial slash wound	5-day course 10	still isolated from	

					(log 8)	min	wound after 8 days	
S.a. unspecified	microwave pulsed	*	930 000 kHz	argon	suspension in artificial saliva (6 log)	7 min (30 min incubation)	5.3 log; 100 % (MTT)	(Seo et al., 2017)
S.a. ATCC 6538	unspecified	25 kV	42 kHz	argon: oxygen	suspension spotted on sliced pastirma pieces (5.8 log)	300 s (100% O_2)	0.8 log	(Gök et al., 2019)
				various ratios		other modes	not effective	
MRSA clinical isolate	plasma jet	10 kV DC	20 kHz	helium	suspension of agar plates	120 s	9 mm	(Namini, Y. N. et al., 2019)
				argon	inhibition zones diameter	120 s	18 mm	
MRSA NCTC 12493	plasma jet	6 kV	20 kHz	oxygen (0.5 %) + helium (99.5 %) (2 L/min)	cell suspension in petri dish (6 log)	120 s	total	(Alkawareek et al., 2014)
S.a. ATCC 29213	plasma jet	17 kV AC	0.06 kHz	air	cell suspension in petri dish (7 log)	60 s	5.6 log	(Yoo et al., 2015)
					then 1 mL transferred to titanium disc	120 s	6.65 log	
S.a. CECT 4459	plasma jet	1 kV	*	air (10 L/min)	pathogen-loaded smoked salmon in sterile petri dish (8-9 log)	15 min	0.5 log	(Colejo et al., 2018)
S.a. ATCC 25923	plasma jet	6 kV	1100 kHz	argon (5 L/min)	suspension in microtiter plate (6 log)	3 min	2 log	(Zhang et al., 2014)
					after treated with 100 pulsed fields (100 µs, 15 kV/cm)	3 min	total	
S.a. ATCC 6538	plasma jet	*	*	*	suspension on agar plates (6.5 log)	1 min	total	(Lunov et al., 2016a)
S.a. unspecified	plasma jet	15-18 kV	30 kHz	helium (2 L/min)	suspension on glass slide (6.3 log)	180 s (16.5 kV)	total	(Liu et al., 2013)
						120 s (18 kV)	total	
S.a. ATCC 25923	plasma jet	8 kV	10 kHz	helium + 2 % oxygen (2 L/min)	suspension on petri dish (6 log)	5 min	3.6 cm (diameter of inhibition zones)	(Cao et al., 2011)
						10 min	8.2 cm	
S.a. CIP 53.154	radio-frequency polarization	*	13 560 kHz	oxygen (0.0005 L/min)	suspension on cover slip (8 log)	120 s (O_2)	7.5 log	(Ben Belgacem et al., 2017)
				argon, nitrogen		120 s (Ar, N_2)	7 log	
S.a. CIP 53.154	radio-frequency polarization	*	13 560 kHz	oxygen (0.0005 L/min)	suspension on glass slides inside	120 s (O_2)	6 log	(Carré et al.,

								2018)
				argon, nitrogen	sealed bags (7 log)	120 s (Ar, N_2)	5.8 log	
MRSA clinical isolate BH1CC	DBD	10 kV DC	25 kHz	air (30 L/min)	biofilm samples on coverslips	90 min (direct exp.)	5.5 log (90 %, MTT)	(Cotter et al., 2011)
					direct exposure to NTP or disinfection by plasma activated air	90 min (activated air)	5.5 log (75 %, MTT)	
S.a. ATCC 25923	DBD	8.5 kV AC	25 kHz	air	suspension (5.2 log) treated before putting on agar	30 s	total	(Usta et al., 2019)
					5-day old biofilm on glass slide	120 s	86% inhibition (MTT)	
S.a. ATCC 25922	DBD reactor	5 kV	37 kHz	air	biofilm on grounded electrode	5 min	5.9 log (99.8 %, MTT)	(Czapka et al., 2018)
MRSA clinical isolate	glow discharge	*	13 560 kHz	helium (1 L/min)	treatment before biofilm formation (5 log)	120 s (0.9 W)	total	(Miletić et al., 2014)
						60 s (1.6 W)	total	
S.a. clinical isolate	plasma jet	6 kV	20 kHz	oxygen (0.5 %) + helium (99.5 %) (2 L/min)	suspension cells (8 log)	240 s	total	(Flynn et al., 2015)
					biofilm in microtiter plate (6.5 log)	360 s	total	
S.a. NCTC 8325	plasma jet	20 kV AC	38 kHz	helium (6.7 L/min)	borosilicate slices with biofilm (6.6 log)	10 min	3.06 log	(Xu et al., 2015)
S.a. ATCC 12598	pulse based plasma generator with plasma nozzle	0.22 kV	*	air	glass coverslips with biofilm (6.5 log)	120 s each day (7 days)	decreased volume, increased porosity (COMSAT analysis)	(Ferrell et al., 2013)
S.a. NCTC 10788	plasma jet	6 kV	20 Hz	helium:oxygen (99.5:0.05) (2 L/min)	biofilm on peg lid of Calgary Biofilm Device (5.5 log)	1 min	total	(Alkawareek et al., 2012b)
S.a. ATCC 9144	SBD	*	*	air	biofilm on PVC coupons (6.3 log)	120 s	3.5 log decrease	(Modic et al., 2017)
S.a. ATCC 25923	DBD micropulsed	31 kV	1.5 kHz	air	plasma activated alginate gels on suspensions	15 min (15 s gel)	total	(Poor et al., 2014)
					plasma activated alginate gels on biofilms	24 h (5 min gel)	20% biofilm viability	

S.a. ATCC 6538	DBD	10 kV	20 kHz	air	plasma activated saline	30 min	2 log	(Oehmigen et al., 2010)
					plasma activated PBS	10-15 min	total	
S.a. ATCC 6538	DBD reactor	20 kV	1 kHz	air	PAW prepared for 5 min mixing with cell suspension (7 log)	24 min	3.5 log	(Laurita et al., 2015)
					PAW prepared for 10 min mixing with cell suspension (7 log)	15 min	3.5 log	
S.a. ATCC 6538	DBD reactor	2-3 kV	25 kHz	air	PAW (30 min) with suspension (5 log)	30 min	total	(Schmidt et al., 2019)
					plasma activated saline (30 min) with suspension (5 log)	30 min	3 log	
S.a. ATCC 29213	micro-hollow cathode	*	*	air (4 L/min)	PAW (30 min) for biofilm treatment	3 h	0.5 log (99.9 %, MTT)	(Chen et al., 2018)
				oxygen (4 L/min)	(4.7 log)	3 h	2.3 log (99 %, MTT)	
S.a. GIM1.441	plasma jet	*	*	air	PAW (60 s) with cell suspension (8 log)	6 min	2.32 log	(Xiang et al., 2019)
S.a. CGMCC 1.2465	plasma microjet	18 kV AC	10 kHz	argon + 2 % oxygen (5 L/min)	PAW (20 mL for 20 min), storage for	10 min (no storage)	total	(Zhang et al., 2013)
					24 h (6 log)	40 min (24 h storage)	total	
S.a. CGMCC 1.2465	plasma microjet	0.4 kV	*	argon + 2 % oxygen (5 L/min)	PAW (electrode above (A) and beneath (B) water) activated for 5-20 min	20 min (PAW A 5 min)	0.4 log	(Tian et al., 2015)
					cells mixed with NTP activated water for 20 min then cultured	20 min (PAW B 5 min)	3.4 log	
						20 min (PAW A 20 min)	1.2 log	
						20 min (PAW B 20 min)	5 log	
S.a. CGMCC 1.2465	plasma jet	*	20 kHz	air (4.33 L/min)	PAW (electrode beneath surface)	0 days storage time (-80 °C storage temperature)	5 log	(Shen et al., 2016)
					different storage temperature and times	30 days (-80 °C)	3.7 log	
						0 days (25 °C)	5 log	
						30 days (25 °C)	0.8 log	

S.a. CGMCC 1.2465	plasma jet	*	10 kHz	argon + 2 % oxygen (5 L/min)	PAW activated for 10 and 20 min for treatment of inoculated strawberries (8 log)	5-15 min (20 min NTP)	1.7-2.3 log	(Ma et al., 2015)
S.a. ATCC 25923, MRSA BBA 1680, MRSA BBA 1683	DBD	20-22 kV	1.5 kHz	bioaerosol of cell suspension	bioaerosol treated, then collected and plated on agar (4 log)	all strains	total	(Vaze et al., 2017)
S.a. ATCC 25923	DBD	32 kV	2.5 kHz	air	contaminated sutures (3 log)	3 min	total	(Ercan et al., 2018)
S.a. ATCC 25923	DBD	30 kV	1.5 kHz	air	plasma activated NAC for treatment of cell suspension or biofilm (7 log)	15 min (3 min activated NAC)	total	(Ercan et al., 2013)
MRSA (USA 100) BAA-1680	DBD	31.4 kV	1.5 kHz	air	plasma activated amino acid solution for treatment of biofilms	methionine, cysteine, glutamine, heparin-activated solutions for 2 min (15 min application)	more than 50 %	(Ercan et al., 2014)

CFU – colony forming units, DBD – dielectric barrier discharge, GRSA – glycopeptide-resistant *S.a.*, MRSA – methicillin resistant *S.a.*, MTT – thiazole salt for cell viability evaluation, NAC – N-acetylcysteine, PAW – plasma activated water, PBS – phosphate buffer saline , RFH – radiofrequency heating, PRSA – pefloxacin-resistant *S.a.*, UV – ultraviolet radiation, US – ultrasound, XTT – method using triazole salt XTT for cell viability determination, * – unspecified parameter

Supplementary Table 3: Overview of NTP inactivation of *Klebsiella pneumoniae* (*K.p.*) strains at various experimental parameters

Microorganism	NTP source				Treated sample (inoculum)	Results		Reference
	Discharge	Voltage	Frequency	Gas (flow rate)		Treatment time	Cell inactivation (CFU)	
K.p. CGMCC 2028	DBD	24 kV	20 kHz	air	cell suspension (8.5 log)	3.5 min	99 %	(Dong et al., 2010)
K.p. blaNDM-1 in a mix/*Klebsiella* spp. SUS9K	DBD	30 kV	40 kHz	air	wastewater from food and leather processing (7 log)	1.5 min	*K.p.* eliminated in both samples	(El-Sayed et al., 2015)
K.p. CGMCC 2028	DBD	13 kV	7 kHz	air	suspension in petri dish	5 min	80 %	(Xiao Yu Dong et al., 2009)
K.p. clinical isolate/*K.p.* ATCC 700324	plasma jet	1-5 kV	1.5 MHz	argon (6 L/min)	inoculated agar plates	3 s	30 mm^2	(Daeschlein et al., 2012)
	DBD	14 kV	0.1-0.4 kHz	air		3 s	271 mm^2	
K.p. ATCC 13883	glow discharge	*	*	air (5 L/min)	inoculated agar plates	10 min	64% inhibition on agar	(Gorbunova, 2019)
K.p. QLR-10 as a part of coconut spoilage	plasma jet	*	2.45 GHz	air (5 L/min)	suspension in petri dish (7 log)	25 min	5 log	(Gabriel et al., 2016)
		*				15 min	6 log	
K.p. CCM 4415	RF barrier discharge	*	13.56 MHz	argon (2 L/min)	inoculated agar plates	5 s	total	(Justan et al., 2014)
K.p. clinical isolate	surface circular plasma	10 kV	2 kHz	air, humidity 90 % (5 L/min)	inoculated steel plates (6 log)	20 min	3.3 log	(Lis et al., 2018)
K.p. clinical isolate	plasma jet	6 kV	20 kHz	oxygen (0.5 %) + helium (99.5 %) (2 L/min)	suspension cells (8 log)	2 min	total	(Flynn et al., 2015)
					biofilm cells (5.5 log)	2 min	total	
K.p. KCOM 2770	DBD plasma jet	15 kV		air (5 L/min)	agar plates (8 log)	10 min	total	(Lee et al., 2019)
					biofilms on titanium disks (8 log)	10 min	55 % (CV)	
K.p. NCTC 13368	SBD	*	*	air	mixed spp. biofilms on PVC coupons (6.3 log)	240 s	4 log	(Modic et al., 2017)

K.p. R-70 (from spoiled tiger nut milk)	plasma jet	*	*	air (39 L/min)	PAW (2-10 min), inoculated tiger nuts (7 log)	15 min (PAW 10 min)	4 log	(Muhammad et al., 2019)
K.p. (NDM1+) BAA-2146	DBD	31.4 kV	1.5 kHz	air	plasma activated amino acid solution for treatment of biofilms	most amino acid solutions activated for 2-3 min (15 min application)	more than 50 %	(Ercan et al., 2014)

CFU – colony forming units, CV – crystal violet, DBD – dielectric barrier discharge, PAW – plasma activated water, RF – radiofrequency, * – unspecified parameter

Supplementary Table 4: Overview of NTP inactivation of *Acinetobacter baumannii* (*A.b.*) strains at various experimental parameters

Microorganism	NTP source				Treated sample (inoculum)	Results		Reference
	Discharge	Voltage	Frequency	Gas (flow rate)		Treatment time	Cell inactivation (CFU)	
A.b. ATCC 17901	DBD	20 kV	*	air	bacteria on agar plates	1 min	inhibition zone diameter of 4 cm	(Atta, 2019)
A.b. ATCC 19606	DBD plasma jet	4.2 or 5.8 kV	*	helium or helium with 3% air	suspension in 96-wells microplates<br>(8 log)	5 min	total	(Parkey et al., 2015)
A.b. clinical isolates	DBD plasma jet	12 kV	10 Hz	helium (2L/min)	suspension in 24-wells microplates or agar plates (3.5 log)	20 min	total	(Svarnas et al., 2019)
A.b. ATCC 19606	plasma jet	2 kV	*	air (8 L/min)	suspension on agar plates (6 log)<br>suspension on pig skin (6 log)	135 s<br>215 s	5.6 log<br>3.8 log	(Heller et al., 2012)
A.b. ATCC 19606	plasma jet	2 kV	*	air (8 L/min)	bacteria on agar plates exposed in different distances (6 log)	135 s (10 mm)	5.5 log	(Kolb et al., 2012)
A.b. multidrug resistant isolate	plasma jet	10 kV	*	air	suspension in Petri dishes (6 log)	10 min	total	(Ruan et al., 2018)
A.b. ATCC 19606	plasma jet (multi-jet)	1.5 kV	8 kHz	air (13 L/min)	bacteria on polyurethane mattress and stainless steel (9 log)	45 s	3 log	(Cahill et al., 2017)
A.b. ATCC 19606	plasma jet<br>(single jet)	2.5 kV	8 kHz	air (12 L/min)	suspension on marmoleum flooring and polyurethane mattress, polypropylene, powder-coated mild steel, and stainless steel (8 log)	1.5 min	4.5-7 log	(Cahill et al., 2014)
A.b. carbapenem-resistant isolate	SBD	10 kV	2 Hz	air (5 L/min), humidity 90 %	biofilm on stainless steel (9 log)	20 min	3.9 log	(Lis et al., 2018)
A.b. NCTC 13304	plasma jet	6 kV	20 kHz	helium:oxygen (99.5:0.05) (2 L/min)	biofilm in 96-wells microplates (8 log)	2 min	total	(Flynn et al., 2015)
A.b. NCTC 13304	plasma jet	6 kV	20 kHz	helium:oxygen (99.5:0.05) (2 L/min)	biofilm in Calgary biofilm device<br>(24-72 h grown) (7 log)	9 min	5 log (24 h grown)	(Flynn et al., 2019)

A.b. ATCC 19606	DBD micropulsed	31 kV	1.5 kHz	air	plasma activated alginate gels (7 log)	15 s	total	(Poor et al., 2014)
A.b. ATCC 19606	DBD	30 kV	1.5 kHz	air	plasma activated NAC for treatment of cell suspension or biofilm (7 log)	15 min (2 min activated NAC)	90%	(Ercan et al., 2013)
A.b. clinical isolate	DBD	31.4 kV	1.5 kHz	air	plasma activated amino acid solutions for treatment of biofilms	most amino acid solutions activated for 2 min (15 min application)	more than 50 %	(Ercan et al., 2014)

CFU – colony forming units, DBD – dielectric barrier discharge, NAC – N-acetylcysteine, PAW – plasma activated water, * – unspecified parameter

Supplementary Table 5: Overview of NTP inactivation of *Pseudomonas aeruginosa* (*P.a.*) strains at various experimental parameters

Microorganism	NTP source				Treated sample (inoculum)	Results		Reference
	Discharge	Voltage	Frequency	Gas (flow rate)		Treatment time	Cell inactivation (CFU)	
P.a. clinical isolates	DBD	10 kV AC	0.05 kHz	air	suspension on glass slides (9 log)	40 min	total	(Mohd Nasir et al., 2016)
	capillary guided corona	13 kV DC	8-15 kHz	air		10 min	total	
P.a. ATCC 27853	DBD (AC)	5.4 kV	50 Hz	oxygen	bacterial suspension in glass Petri dishes (8 log)	10 min	total	(Sohbatzadeh et al., 2010)
P.a. unspecified	DBD (AC)	32 kV	1 kHz	mixture of ambient air and CO_2 in the ratio 25:1	treated LDPE samples in suspension of *P.a.* - impact on biodegradation of LDPE	5 min	increased biodegradation of LDPE	(Scally et al., 2018)
P.a. 103 + 2 clinical isolates	microwave torch (MicroPlaSter β device)	18 V DC	2.45 GHz	argon	suspension on agar plate (5 log) or biofilm on glass cover slips	5 min	total	(Ermolaeva et al., 2011)
P.a. ATCC 27853	tube plasma reactor (microwave)	45 V	9.8 GHz	argon (0.2 L/min)	contaminated PET sheets (inoculum unspecified)	1 min	total	(Yang et al., 2009)
P.a. - 30 isolates	needle plasma system (NPS)	20 kV	*	argon (0.5 L/min)	bacterial suspension in petri dishes (23 log)	60 min	3.1 log	(Mohammed and Abbas, 2018)
P.a. unspecified	plasma needle	7.5 kV	28 kHz	argon (1 L/min)	bacterial suspension in 96-well microplate (8 log)	40 s	total	(Humud, 2019)
P.a. clinical isolate	plasma needle (AC)	8 kV	*	argon (5 L/min)	bacterial suspension in petri dishes (8.2 log)	5 min	total	(Abbas et al., 2017)
P.a. ATCC 27853	plasma needle	*	13.56 MHz	helium (1 L/min)	bacterial suspension (8.2 log)	160 s	total	(Puač et al., 2014)
P.a. O1 (ATCC BAA-47)	plasma jet	6 kV	20 kHz	helium:oxygen (99.5:0.05)	biofilm on peg lid of Calgary Biofilm Device, polycarbonate coupons (6.2 log)	4 min	4 log	(Alkawareek et al., 2012b)

			40 kHz	(2 L/min)		4 min	total	
P.a. O1	plasma jet	6 kV	20 kHz	helium:oxygen (99.5:0.05) (2 L/min)	cell suspension (6.5 log)	2 min	total	(Alkawareek et al., 2014)
P.a. O1	plasma jet	6 kV	20 kHz	helium:oxygen	suspension in 96-wells microplates	10 min	total inhibition of endotoxin concentration	(Barakat et al., 2019)
P.a. ATCC 15442	plasma jet, DBD type (AC)	*	*	helium<br>(2 or 4 L/min)	bacteria on agar plates	3 min	inhibition zone 100 mm^2	(Nishime et al., 2017)
P.a. O1	plasma jet	7 kV	8 kHz	mixture of helium, oxygen, and nitrogen	mouse vaccinated by bacterial suspension (9 log)	10 min each day, 14 d	6 log	(Muhammad et al., 2019)
P.a. ATCC 27853	plasma jet	*	*	*	bacteria on agar plates	1 min	total	(Lunov et al., 2016a)
P.a. unspecified	plasma jet	*	*	air, helium, nitrogen	unspecified	1 min	total	(Lunov et al., 2016b)
P.a. 14	plasma jet	*	13.3 MHz modulated at 20 kHz	argon, argon mixed with 1% oxygen, and argon with 1% air	bacterial suspension in 96-well microplate (6-7 log)	15 min	total	(Kondeti et al., 2018)
P.a. ATCC 27853	plasma jet	2 kV	*	air (8 L/min)	bacteria on agar plates or pig skin	135 s	5.6 log decrease for agar plates	(Heller et al., 2012)
P.a. ATCC 27853	plasma jet	2 kV	*	air (8 L/min)	bacteria on agar plates exposed in different distances	111 s (10 mm)	5.6 log decrease for agar plates	(Kolb et al., 2012)
P.a. O1, *P.a.* AK44, P.a. 1.1.A1 and P.a. 19G12	oxygen glow discharge plasma (DC)	1.5 kV	*	oxygen	adhesion on oxygen-plasma treated poly(vinyl chloride) (PVC) from endotracheal intubation devices<br>(5-7 log)	2 min	60-70 % reduction	(Triandafillu, 2003)
P.a. unspecified	air glow discharge plasma	8-11.5 kV	5-10 kHz	air	bacteria on polypropylene (7.3 log)	0.5 min	6 log	(Gadri et al., 2000)
P.a. O1	commercially available inductively coupled Atomflo 300 reactor	*	13.56 MHz	He (20.4 L/min), a secondary gas flow (N_2) of 0.15 L/min	biofilms grown on borosilicate coupons (6 log)	5 min	total	(Zelaya et al., 2010)
P.a. O1	DBD plasma reactor (AC)	8 kV	50 Hz	air (80 % humidity;	biofilm on stainless-steel 316L (6.5 log)	30 min	total	(Soler-Arango et al., 2019)

				1 L/min)				
P.a. ATCC 27853	DBD	80 kV	50 Hz	air	biofilm in 96-wells microplates or PET membrane (7 log)	5 min	biofilm unaffected (CFU), pyocyanin and elastase Las B production was decreased	(Ziuzina et al., 2015)
P.a. ATCC 27853	DBD	80 kV	50 Hz	air	biofilm in 96-wells microplates or glass coverslips (6.6 log)	5 min	70 % (XTT)	(Ziuzina et al., 2014)
P.a. CIP A22	microwave chamber	*	2.45 GHz	two mixtures of oxygen and nitrogen (5% O_2–95% N_2 or 15% O_2–85% N_2)	biofilm on disks made of a hydroxyapatite-coated titanium alloy (5.8 log)	60 min	total	(Ben Belgacem et al., 2016)
P.a. SG81	plasma jet KINPen 09 (DC)	2-6 kV	1.1 MHz	argon or argon/oxygen mixture (5 L/min)	biofilm in 96-wells microplates (5.5 log)	5 min	total	(Matthes et al., 2013a)
P.a. O1	plasma jet	6 kV	20 Hz	helium:oxygen (99.5:0.05) (2 L/min)	biofilm on peg lid of Calgary Biofilm Device (6 log)	10 min	total	(Alkawareek et al., 2012a)
P.a. ATCC 27853 + 3 isolates from water tank	plasma jet (microwave generator)	*	2.45 GHz	air (5 L/min)	biofilm on stainless-steel 316 and 304<br>(5 log)	1.5 min	total	(Gabriel et al., 2016)
P.a. 14	plasma jet	6 kV	20 kHz	helium:oxygen (99.5:0.05) (2 L/min)	biofilm in 96-wells microplates (6 log)	6 min	total	(Flynn et al., 2015)
P.a. O1	plasma jet (kINPen med)	*	1.82 MHz	argon (3.1 L/min)	dried bacterial suspension or biofilm on stainless steel coupons (4.5 log)	10 min	3.5 log	(Mai-Prochnow et al., 2016)
P.a. ATCC 9027	plasma jet (kINPen med)	*	1.82 MHz	argon (3.1 L/min)	biofilm on glass or stainless steel coupons (6 log)	5 min	total	(Mai-Prochnow et al., 2015)
P.a. SG81	plasma pen (kINPen 09)	4 kV	1.82 MHz	argon (5 L/min)	biofilm on polystyrene microplates and silicone swatches (8 log)	1 min	4 log	(Hübner et al., 2010)
P.a. O1	plasma jet	10 kV	25 kHz	helium (2 L/min)	early biofilm on polycarbonate 0-8 h<br>late biofilm 12-24 h (both 10 log)	5 min<br>5 min	4-5 log<br>2 log	(Patenall et al., 2018)
P.a. O1	plasma jet	10 kV	1 kHz	helium (1 L/min)	biofilm on titanium coupons (8 log)	chlorhexidine + 15 min	4.5 log	(Gupta et al., 2017)

						15 min + chlorhexidine	total	
P.a. O1	plasma jet	*	13.56 MHz	He (20.4 L/min), a secondary gas flow (N_2) of 0.135 L/min	biofilm on borosilicate surface in a continuous culture system (8 log)	0-30 min	8 log decrease (total); 30 min	(Vandervoort and Brelles-Mariño, 2014)
P.a. DBM 3777	point-to-point DC cometary discharge with a metallic grid	5 kV	*	air	biofilm on Ti-6Al-4V titanium alloy (OD_{600nm} 0.6)		80 % (CV); 50 % (MTT)	(Paldrychová et al., 2019)
P.a. ATCC 10145							50 % (CV); 50 % (MTT)	
P.a. ATCC 15442							50 % (CV); 50 % (MTT)	
P.a. clinical isolates							not effective	
P.a. DBM 3777	point-to-point DC cometary discharge with a metallic grid	5 kV	*	air	biofilm on Ti-6Al-4V titanium alloy (OD_{600nm} 0.6) treated with NTP in combination with antibiotics	30 min + gentamicin	90 % (CV); total (MTT)	(Paldrychová et al., 2020)
P.a. DBM 3081						60 min + gentamicin	70 % (CV); 70 % (MTT)	
P.a. ATCC 10145						30 min + gentamicin	80 % (CV); 90 % (MTT)	
P.a. ATCC 15442						15 min + gentamicin	total (CV); total (MTT)	
P.a. ATCC BAA-2108	RF plasma source	1 kV	4.6 MHz	helium (1–3 L/min)	biofilm on borosilicate (6 log) slides in 24-well culture plates	2 min	4 log (+ altered membrane)	(Brun et al., 2018)
P.a. ATCC 29260	pulse based plasma generator with plasma nozzle	0.22 kV	*	air	biofilm on glass coverslips (6.5 log)	0.5-2 min	none	(Ferrell et al., 2013)
P.a. SG81	SBD	8-13 kV	20-30 Hz	air	biofilm on polycarbonate discs (7 log)	10 min	4-7 log	(Matthes et al., 2013b)
P.a. O1	SBD	*	*	air	biofilm on PVC coupons (6.8 log)	120 s	4 log	(Modic et al., 2017)
P.a. SG81	SBD	4 kV	30 Hz	argon	biofilms on polycarbonate disks (8 log)	5 min	4.9 log	(Matthes et al., 2014)
				argon + 1% oxygen		5 min	3 log	

				argon (80% humidity)		5 min	2.7 log	
commercially available AHL molecules (produced usually by *P.a.*)	plasma jet	6 kV	20 kHz	helium:oxygen (99.5:0.05) (2 L/min)	exposed AHL solution (20 µL)	0-4 min	degradation of AHL molecules	(Flynn et al., 2016)
P.a. ATCC 15442	plasma jet KINPen 09 (DC) - tissue tolerable plasma	*	1.8 MHz	argon; argon:oxygen (5 L/min)	extracted eyes from pigs inoculated by bacteria (8 log)	pulsed mode of total duration 200 µs	2.5 log (10 min plasma)	(Hammann et al., 2010)
P.a. unspecified	DBD	30 kV	1.5 kHz	air	plasma activated NAC for treatment of cell suspension or biofilm (7 log)	15 min (3 min activated NAC)	total	(Ercan et al., 2013)

AC – alternating current, AHL – N-acyl homoserine lactones, CFU – colony forming units, DBD – dielectric barrier discharge, DC – direct current, LDPE – low-density polyethylene, MTT – thiazole salt for cell viability evaluation, NAC – N-acetylcysteine, OD – optical density, PAW – plasma activated water, PBS – phosphate buffer saline, PET – polyethylene terephthalate, PVC – polyvinylchloride, RF – radiofrequency, SBD – surface barrier discharge, XTT – method using triazole salt XTT for cell viability determination, * – unspecified parameter

Supplementary Table 6: Overview of NTP inactivation of *Enterobacter spp.* strains at various experimental parameters

Microorganism	NTP source				Treated sample (inoculum)	Results		Reference
	Discharge	Voltage	Frequency	Gas (flow rate)		Treatment time	Cell inactivation (CFU)	
E.c. ATCC 35030	DBD plasma jet	4.2 or 5.8 kV	*	helium or helium with 3% air	suspension in 96-wells microplates (7 log)	3 min (He)	total	(Parkey et al., 2015)
E.ae. BMW/2E	DBD	30 kV	40 kHz	air	wastewater from food and leather processing (7 log)	30 s	total elimination in the mix	(El-Sayed et al., 2015)
E.ae. IAM1183	glow discharge plasma jet	170 kV	*	helium (15 L/min)	suspension on stainless steel (7 log)	3 min	total	(Lu et al., 2011)
E.ag. K-7	DBD	0.03 kV	*	air	suspension in petri dish	4 min	more than 90 %	(Ren et al., 2012)
E.c. clinical isolate	plasma jet	6 kV	20 kHz	oxygen (0.5 %) + helium (99.5 %) (2 L/min)	suspension cells (8 log)<br>biofilm in microtiter plate (6 log)	45 s<br>2 min	total<br>total	(Flynn et al., 2015)
E.c. unspecified	plasma jet (kINPen med)	*	1.82 MHz	argon (3.1 L/min)	biofilm on stainless steel (6 log)	10 min	3.5 log	(Mai-Prochnow et al., 2016)
Enterobacter spp.	SF6 plasma	*	*	*	plasma treated polymers (PET, PVC, PE)	1 min	total	(Tiwari and Chaturvedi, 2018)

DBD – dielectric barrier discharge, *E.c. – Enterobacter cloacae, E. ae. – Enterobacter aerogenes, E.ag. – Enterobacter agglomerans*, PE – polyethylene, PET – polyethylene terephthalate, PVC – polyvinylchloride, SF6 – sulphur hexafluoride
